# QTL‐seq for rapid identification of candidate genes for 100‐seed weight and root/total plant dry weight ratio under rainfed conditions in chickpea

**DOI:** 10.1111/pbi.12567

**Published:** 2016-05-26

**Authors:** Vikas K. Singh, Aamir W. Khan, Deepa Jaganathan, Mahendar Thudi, Manish Roorkiwal, Hiroki Takagi, Vanika Garg, Vinay Kumar, Annapurna Chitikineni, Pooran M. Gaur, Tim Sutton, Ryohei Terauchi, Rajeev K. Varshney

**Affiliations:** ^1^ International Crops Research Institute for the Semi‐Arid Tropics (ICRISAT) Hyderabad India; ^2^ Department of Genetics Osmania University Hyderabad India; ^3^ Iwate Biotechnology Research Center Kitakami Iwate Japan; ^4^ South Australian Research and Development Institute Adelaide Australia; ^5^ School of Agriculture, Food and Wine The University of Adelaide Adelaide Australia; ^6^ School of Plant Biology and Institute of Agriculture The University of Western Australia Crawley WA Australia

**Keywords:** chickpea, resequencing, trait mapping, seed weight, root ratio, SNP‐index

## Abstract

Terminal drought is a major constraint to chickpea productivity. Two component traits responsible for reduction in yield under drought stress include reduction in seeds size and root length/root density. QTL‐seq approach, therefore, was used to identify candidate genomic regions for 100‐seed weight (100SDW) and total dry root weight to total plant dry weight ratio (RTR) under rainfed conditions. Genomewide SNP profiling of extreme phenotypic bulks from the ICC 4958 × ICC 1882 population identified two significant genomic regions, one on CaLG01 (1.08 Mb) and another on CaLG04 (2.7 Mb) linkage groups for 100SDW. Similarly, one significant genomic region on CaLG04 (1.10 Mb) was identified for RTR. Comprehensive analysis revealed four and five putative candidate genes associated with 100SDW and RTR, respectively. Subsequently, two genes (*Ca_04364* and *Ca_04607*) for 100SDW and one gene (*Ca_04586*) for RTR were validated using CAPS/dCAPS markers. Identified candidate genomic regions and genes may be useful for molecular breeding for chickpea improvement.

## Introduction

Chickpea is the second most important grain legume crop, cultivated predominantly by resource poor farmers in arid and semi‐arid regions of the world. Annual global production of chickpea is around 13.1 million tons from 13.5 Mha (FAOSTAT, 2013). India is the major chickpea producing country, producing 67% of the world's chickpea in 2013 (FAOSTAT, 2013). Besides being an important source of protein for millions of people in developing countries, particularly in South Asia, it is a rich source of fibre, minerals (phosphorus, calcium, magnesium, iron and zinc) and β‐carotene. In addition, chickpea can obtain over 70% of its nitrogen requirement through symbiotic nitrogen fixation (SNF) by fixation of 140 kg/ha of atmospheric nitrogen to the soil (Flowers *et al*., 2010; Gaur *et al*., 2012), although chickpea has high calorific value (364 kcal/100 g after soybean 446 kcal/100 g; Akibode and Maredia, 2011) and high protein content (18%–25%). However, the average per capita availability of chickpea decreased by 6% across the world and 14% especially in India between 1990 and 2008 due to increase in population growth. In addition, several biotic and abiotic stresses have also been limiting the chickpea productivity especially in India, which happens to be the largest producer and consumer as well. Nevertheless, with the expansion in international trade for chickpea, several developing countries (e.g. Ethiopia), as well as developed countries (e.g. Australia, Canada), have been emerging as major exporters of chickpea to India.

Development and adoption of improved varieties with higher yield will enhance per capita consumption, thereby reducing the number of malnourished people across the world, especially in developing countries. In this direction, in addition to conventional breeding strategies, genomics approaches are being deployed in recent years to identify genomic regions that confer resistance/tolerance for different stresses. Both biparental mapping and association mapping approaches have been utilized to dissect complex traits in chickpea. However, to date, the limited number of trait mapping studies has mainly focused on mapping biotic stresses such as *Fusarium* wilt (Gowda *et al*., 2009; Sabbavarapu *et al*., 2013), ascochyta blight (Udupa and Baum, 2003; Kottapalli *et al*., 2008) and botrytis grey mould (Anuradha *et al*., 2011), as well as agronomically important traits (Das *et al*., 2015; Gowda *et al*., 2011). Efforts have also been made to dissect complex abiotic stresses such as salinity (Vadez *et al*., 2011) and drought tolerance (Hamwieh *et al*., 2013; Jaganathan *et al*., 2015; Kale *et al*., 2015; Rehman *et al*., 2011; Thudi *et al*., 2014; Varshney *et al*., 2014a) with an emphasis on understanding the performance of agronomic or physiological traits.

Recently, Varshney *et al*. (2014a) reported a genomic region on linkage group 4 in chickpea (CaLG04) referred as ‘*QTL‐hotspot*’ for drought responsive traits. This genomic region explains highest phenotypic variation (PVE) of 58.20% for 100SDW, an important trait that has relevance to yield and 16.67% PVE in case of root dry weight/total plant dry weight ratio (RTR) that has relevance to drought tolerance (Kashiwagi *et al*., 2006). Owing to the large size of this genomic region, the region was refined using a genotyping‐by‐sequencing (GBS) approach (Jaganathan *et al*., 2015). As a result, the ‘*QTL‐hotspot*’ region was narrowed to 14 cM (~3 Mb) and candidate genes prioritized for analysis (Kale *et al*., 2015).

In recent years, advances in next generation sequencing technologies (NGS) have seen the rapid decrease in cost of sequencing, enabling the cost‐effective application of genomics in crop improvement possible (Varshney *et al*., 2014b). The advantage of identifying large numbers of SNPs through resequencing has made it possible to locate and refine candidate genomic regions more efficiently compared to traditional QTL mapping approaches (Chen *et al*., 2014; Kale *et al*., 2015; Qi *et al*., 2014; Xu *et al*., 2013). Bulk segregant analysis (BSA) is a rapid method to identify markers linked to the trait of interest (Michelmore *et al*., 1991) and when combined with NGS technologies can be used as a fast track approach to locate candidate genomic regions more rapidly. This approach is known as QTL‐seq and involves the selection of 20–50 lines with extreme phenotypic values, pooling in equivalent concentration followed by sequencing of pools for downstream sequence analysis (Takagi *et al*., 2013). The QTL‐seq approach has been used successfully to map candidate genomic regions for early flowering in cucumber (Lu *et al*., 2014), fruit weight and locule number in tomato (Illa‐Berenguer *et al*., 2015) and 100‐seed weight in chickpea (Das *et al*., 2015).

With an objective of identifying candidate genomic regions responsible for 100SDW and RTR, the QTL‐seq approach was adopted. We were able to precisely localize the genomic regions for the two target traits and identify five genic SNPs in four candidate genes for 100SDW and six genic SNPs in five candidate genes for RTR. The involvement of candidate genes was further validated through co‐segregation analysis in the entire RIL population derived from ICC 4958 × ICC 1882.

## Results

### Extreme bulks for 100SDW and RTR

Based on phenotyping data generated earlier for the ICC 4958 × ICC 1882 mapping population (Varshney *et al*., 2014a), two extreme bulks each for 100SDW and RTR were prepared and subjected to the QTL‐seq pipeline as shown in Figure S1 for supplementary figures. In brief, phenotypic data for 100SDW showed a skewed segregation towards the tolerant parent ICC 4948 (Figure S2), with trait values of RILs ranging between 12.24 and 30.80 g [values for parental lines: 30.65 g ± 2.65 (ICC 4958); 14.03 g ± 3.21 (ICC 1882)]. Fifteen RILs with high 100SDW (25.27–30.80 g) and 15 RILs with low 100SDW (12.24–13.59 g) were selected to prepare the two extreme bulks. The phenotyping data for RTR among RILs showed Gaussian segregation (Figure S3). The phenotypic values among RILs for RTR across three seasons varied from 23.40% to 39.95% [values for parental lines: 31.83% ±0.02 (ICC 4958); 26.38% ±1.88 (ICC 1882)]. To prepare the high RTR and low RTR bulks, a total of 15 RILs with high RTR (ranged from 33.18% to 39.95%) and 15 RILs with low RTR (ranged from 23.40% to 25.84%) were selected, respectively (Figure S4).

### Whole‐genome resequencing and mapping of reads

Five Illumina libraries (two for 100SDW bulks, two for RTR bulks and one for ICC 4958, the drought tolerant parent) were constructed and subjected to whole‐genome resequencing using Illumina MiSeq. In total, 38.46 million paired‐end (PE) reads (17.79 million reads for high and 20.67 million reads for low 100SDW bulks, respectively) for 100SDW and 35.52 million PE reads (17.95 for high and 17.57 million reads for low RTR bulks, respectively) for RTR were generated. A total of 29.29 million PE reads were generated for ICC 4958 (Table 1). Alignment of the PE reads generated from ICC 4958 to the reference genome assembly of chickpea (Varshney *et al*., 2013) resulted in an average depth of 6.52X and 96.35% genome coverage, allowing us to develop a reference‐based assembly of ICC 4958 (hereafter designated as ICC 4958 assembly). Mapping of the PE reads generated from extreme bulks to the developed ICC 4958 assembly for 100SDW resulted in 3.18X and 3.17X sequencing depth and 81.73% and 81.53% coverage for high 100SDW and low 100SDW bulks, respectively. Similarly, for high RTR and low RTR bulks, we obtained alignment of 3.21X and 3.17X sequencing depth and 81.94% and 81.05% coverage, to the ICC 4958 assembly, respectively. Of the 6001 polymorphic SNPs identified between the high and low bulks of 100SDW, 1516 were homozygous (Table S1 for supplementary tables). While in the case of RTR, 3792 were homozygous among 7318 SNPs identified between bulks (Table S2).

**Table 1:** Sequencing of parental lines and bulks and mapping of sequence reads

Genotypes	Number of bulked lines	Number of reads	Unmapped read (%)	Genome coverage (%)	Average depth (X)
ICC 4958^a^	–	29299428	0.39	96.35	6.52
High 100SDW bulk^b^	15	17795370	0.29	81.73	3.18
Low 100SDW bulk^b^	15	20670924	0.29	81.53	3.17
High RTRbulk^b^	15	17954432	0.28	81.94	3.21
Low RTRbulk^b^	15	17574432	0.29	81.05	3.17

a ICC 4958 short reads were aligned to the publicly available chickpea genome of CDC Frontier (Varshney *et al*., 2013), a *kabuli* chickpea variety.

b The short reads of bulks were aligned to the ICC 4958 assembly developed by replacement of SNPs between ICC 4958 and CDC Frontier.

### Candidate genomic regions for 100SDW and RTR

To identify candidate genomic region(s) responsible for differences in 100SDW and RTR, between the low and high RIL bulks, we compared the ‘SNP‐index’ between them. Focusing on a particular SNP position of the genome, the SNP‐index is calculated as a ratio of PE short reads aligned to that position with a nucleotide different from that of the reference sequence. The SNP‐index represents frequencies of parental alleles in the population of bulked individuals. In this case, the ICC 4958 genome was used as the reference, where a SNP‐index of 1 indicates that reads in the population are derived only from the ICC 1882 genome, whereas SNP‐index = 0 indicates the reads are derived only from ICC 4958. A SNP‐index of 0.5 indicates an equal genome contribution from both parents. A significant deviation from SNP‐index of 0.5 could indicate the contribution of that SNP to the phenotypic difference observed in the bulks (Abe *et al*., 2012).

SNP‐index values across the genome were calculated and sliding window averages of 2 Mb interval with 10 kb increments were plotted for high and low bulks. To facilitate detection of differences in SNP‐indices between high and low bulks, we plotted ∆SNP‐index with a statistical confidence interval. With a statistical significance of *P* < 0.05, significant genomic positions were identified.

**Figure 1:**
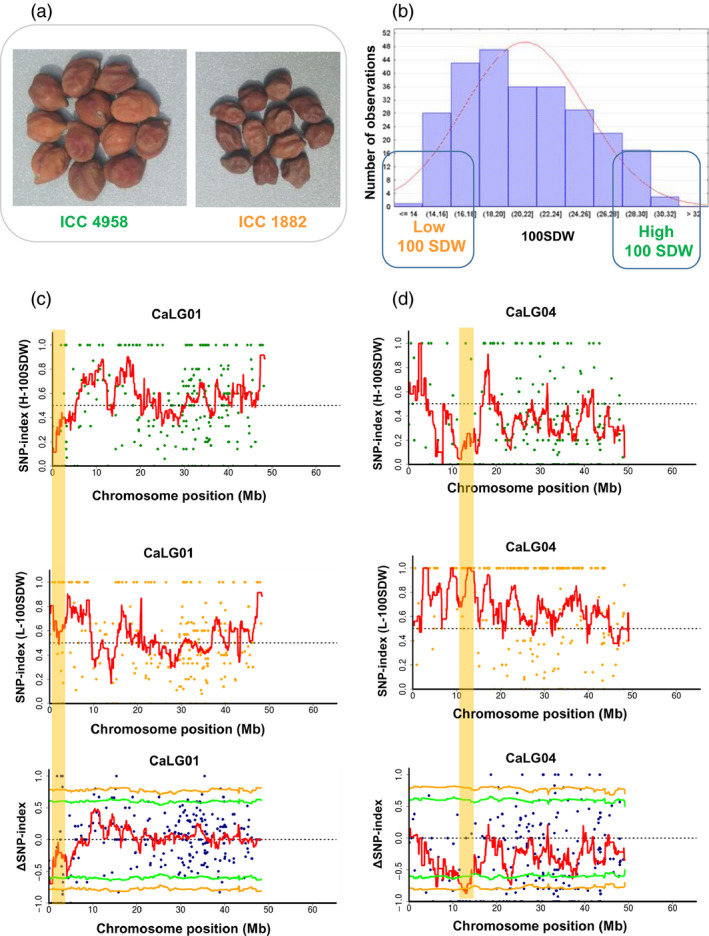
QTL‐seq approach adopted for mapping genomic regions responsible for 100SDW. (a) Image shows the morphological difference of ICC 4958 (tolerant parent with large seed size) and ICC 1882 (sensitive parent with small seed size) (b) Frequency distribution of 100SDW of 262 RILs based on five years of mean data. The DNA of 15 RILs with extreme phenotypes (high and low 100SDW) was used to develop high and low 100SDW bulks. (c) SNP‐index plot of high 100SDW bulk (top), low 100SDW bulk (middle) and ΔSNP‐index plot (bottom) of chromosome 1. The significant genomic regions are highlighted in shaded colour (3.07–4.15 Mb). (d) SNP‐index plot of high 100SDW bulk (top), low 100SDW bulk (middle) and Δ SNP‐index plot (bottom) of chromosome 4. The significant genomic regions are highlighted in shaded colour (11.12–13.82 Mb). The statistical confidence interval under the null hypothesis of no QTLs is presented in the graphs (orange, *P* < 0.01 and green *P* < 0.05).

Sequence analysis for 100SDW revealed two genomic regions on two linkage groups (CaLG01 and CaLG04) (Figures 1 and S5–S7). In both regions, alleles from ICC 4958 contributed to higher trait values and those from ICC 1882 to lower trait values. A genomic region spanning 1.08 Mb (3.07–4.15 Mb) on CaLG01 showing significant (*P* < 0.05) deviation from equal inheritance of the two parental genomes had five SNPs with ∆SNP‐index = −1 (Table S3). However, none of the SNPs was from predicted genic regions. The lower number of SNPs may be due to the narrow candidate region and/or low coverage. Further, the second 2.70 Mb region (11.12–13.82 Mb) on CaLG04 harboured 21 SNPs with ∆SNP‐index = −1. Of these 21 SNPs, five SNPs were present in four putative genes (*Ca_04364*, *Ca_04600*, *Ca_04602* and *Ca_04607*; Table 2). Of these five genic SNPs, four were in intronic region and one SNP (Ca_04607_13822383) in the exon of a predicted gene *Ca_04607*, associated with transmembrane protein, causing a nonsynonymous substitution from valine (ICC 4958) to isoleucine (ICC 1882).

**Table 2:** Identification of SNPs in putative candidate genes for 100‐seed weight (100SDW)

Linkage group	Gene	Position	ICC 4958 allele	High 100SDW bulk allele	SNP‐index (high 100SDW bulk)^a^	Low 100SDW bulk allele	SNP‐index (low 100SDW bulk)^b^	Δ SNP‐index^c^	SNP effect	Function
CaLG04	*Ca_04364*	11311944	A	A	0	C	1	−1	Intron	Cell division protein kinase
CaLG04	*Ca_04600*	13760326	C	C	0	T	1	−1	Intron	Uncharacterized protein
CaLG04	*Ca_04602*	13780146	C	C	0	G	1	−1	Intron	Random slug protein
CaLG04	*Ca_04607*	13822383	G (Gtc/V)^d^	G (Gtc/V)^d^	0	A (Atc/I)^d^	1	−1	Exon	Transmembrane protein
CaLG04	*Ca_04607*	13822453	A	A	0	G	1	−1	Intron	Transmembrane protein

a SNP‐index of high 100SDW bulk was calculated based on the allele calls and read depth in comparison with ICC 4958 reference assembly.

b SNP‐index of low 100SDW bulk was calculated based on the allele calls and read depth in comparison with ICC 4958 reference assembly.

c Δ SNP‐index of each SNP positions was calculated using following formula: Δ SNP‐index = SNP‐index of high 100SDW bulk—SNP‐index of low 100SDW bulk.

d Value in parenthesis indicates the codon change due to SNP/Code for changed amino acids V, valine; I, isoleucine.

**Figure 2:**
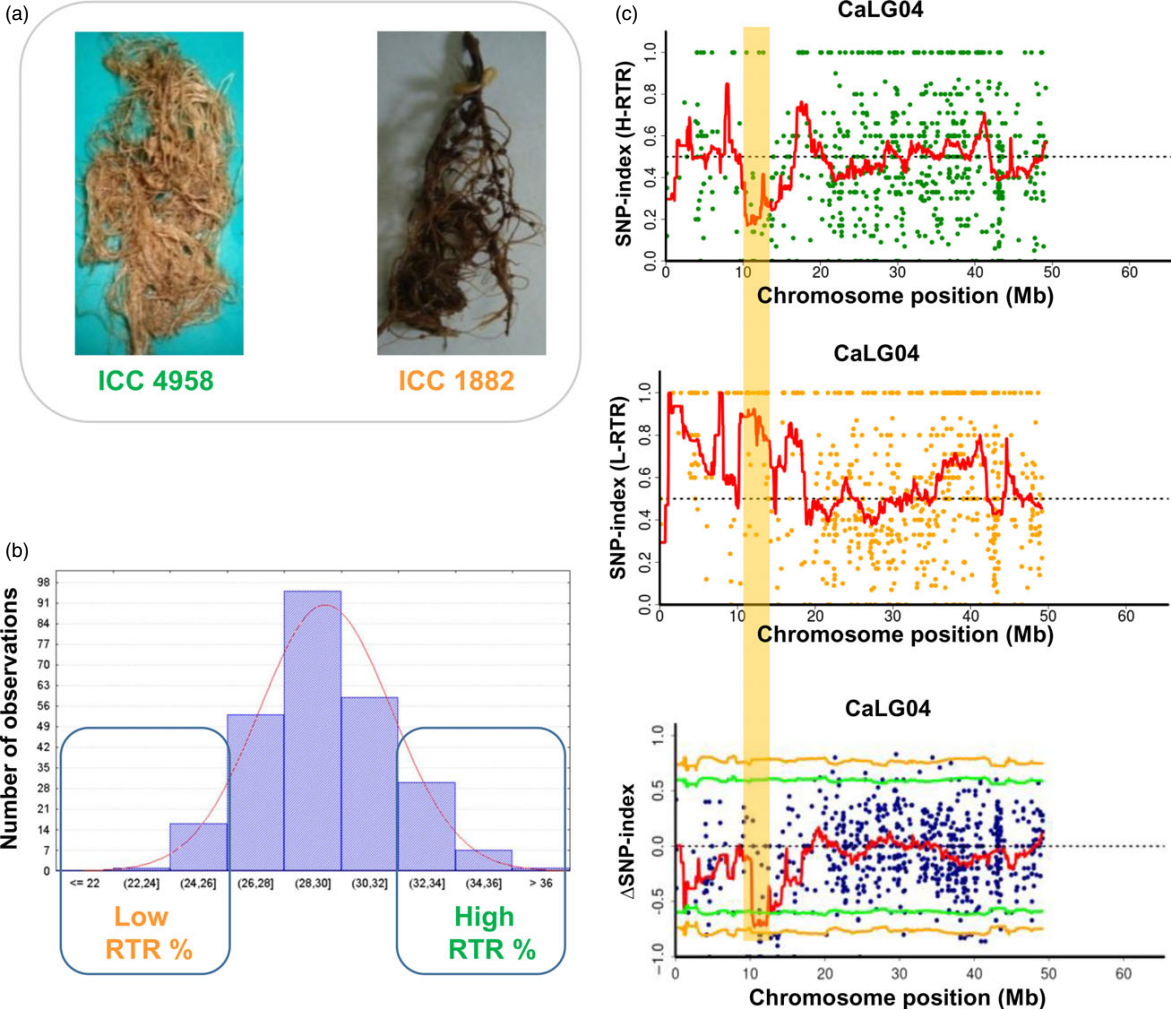
QTL‐seq approach adopted for mapping genomic regions responsible for RTR. (a) Morphological differences between drought tolerant parent ICC 4958 (with larger root system) and ICC 1882 (sensitive parent with smaller root system) (b) Frequency distribution of RTR of 262 RILs based on 2 years mean data. The DNA of 15 RILs with extreme phenotypes (high and low RTR) was used to develop high and low RTR % bulks. (c) SNP‐index plot of high RTR bulk (top), low RTR bulk (middle) and ΔSNP‐index plot (bottom) of chromosome 4 with statistical confidence interval under the null hypothesis of no QTLs (orange, *P* < 0.01 and green *P* < 0.05). The significant genomic regions are highlighted in orange shaded colour (12.73–13.83 Mb).

Similarly, sequence analysis of low and high RTR bulks for the identified 1.10 Mb region (12.73–13.83 Mb) on linkage group 4 (CaLG04) revealed 26 SNPs with ∆SNP‐index = −1, suggesting a biased inheritance of parental genomes in the two populations (Figures 2 and S8–S10). The high RTR bulk showed SNP‐index = 0 indicating that high trait value alleles were inherited from the tolerant parent ICC 4958. By contrast, low RTR bulk at these 26 positions possesses SNP‐index = 1, indicating that their alleles were derived from the susceptible parent ICC 1882 (Table S4). Of 26 SNPs, six SNPs were found to occur in five predicted genes (*Ca_04493*, *Ca_04586*, *Ca_04592*, *Ca_04595* and *Ca_04602*). Interestingly, two SNPs (at 13666705 bp for synonymous change and 13666728 bp for T to I amino acid change) were identified in the exon of a putative gene, *Ca_04586* predicted to be coding for cytochrome P450 monooxygenase (Table 3).

**Table 3:** Identification of SNPs in putative candidate genes for total dry root weight to total plant dry weight ratio (RTR)

Linkage group	Gene	Position	ICC 4958 allele	High RTR bulk allele	SNP‐index (high RTR bulk)^a^	Low RTR bulk allele	SNP‐index (low RTR bulk)^b^	Δ SNP‐index^c^	SNP effect	Function
CaLG04	*Ca_04493*	12737206	T	T	0	G	1	−1	Intron	Uncharacterized protein
CaLG04	*Ca_04586*	13666705	C (tcC/S)^d^	C (tcC/S)^d^	0	T (tcT/S)^d^	1	−1	Exon	Cytochrome P450 monooxygenase
CaLG04	*Ca_04586*	13666728	C (aCa/T)^d^	C (aCa/T)^d^	0	T (aTa/I)^d^	1	−1	Exon	Cytochrome P450 monooxygenase
CaLG04	*Ca_04592*	13708182	T	T	0	C	1	−1	Intron	Thiamine thiazole synthase
CaLG04	*Ca_04595*	13716902	A	A	0	G	1	−1	Intron	Imidazoleglycerol‐phosphate dehydratase
CaLG04	*Ca_04602*	13781245	G	G	0	C	1	−1	Intron	Random slug protein

a SNP‐index of high RTR bulk was calculated based on the allele calls and read depth in comparison with ICC 4958 reference assembly.

b SNP‐index of low RTR bulk was calculated based on the allele calls and read depth in comparison with ICC 4958 reference assembly.

c Δ SNP‐index of each SNP positions was calculated using following formula: Δ SNP‐index=SNP‐index of high RTR bulk—SNP‐index of low RTR bulk.

d Value in parenthesis indicates the codon change due to SNP/Code for changed amino acids S, serine; T, threonine; and I, isoleucine.

### Validation of identified genomic regions

**Figure 3:**
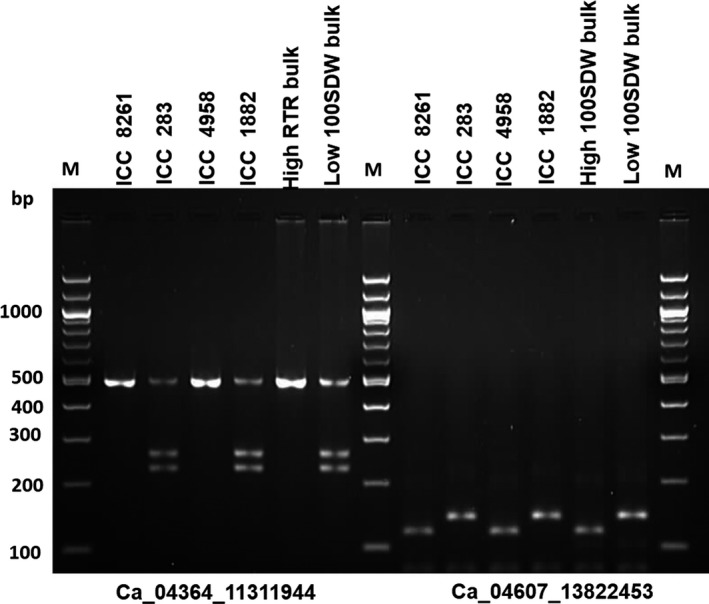
Validation of candidate gene‐based markers for 100SDW. Two gene‐based markers Ca_04364_11311944 (CAPS) and Ca_04607_13822453 (dCAPS) associated with 100SDW showed clear polymorphism between ICC 4958 and ICC 1882 after digestion with *AluI* and *MseI* restriction enzyme, respectively. The PCR amplicons also correspond to the high and low 100SDW bulk along with other two parental lines (ICC 8261—high seed weight parent and ICC 283—low seed weight parent) of different mapping population.

**Figure 4:**
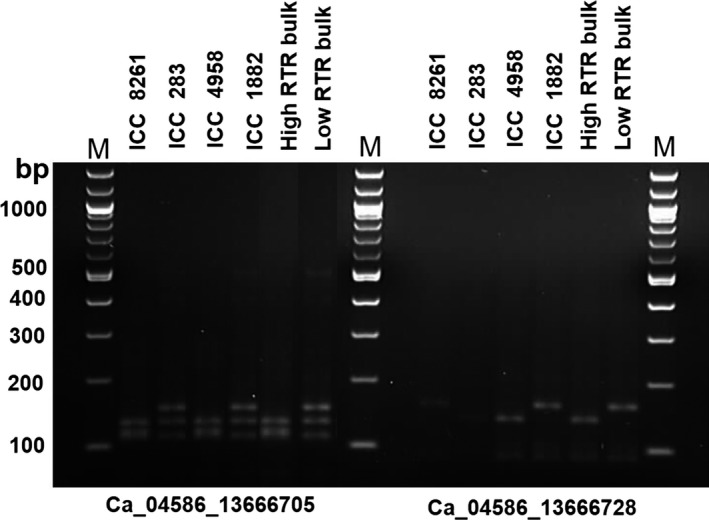
Validation of candidate gene‐based markers for RTR. Two gene‐based markers Ca_04586_13666705 (dCAPS) and Ca_04586_13666728 (dCAPS) associated with RTR showed clear polymorphism between ICC 4958 and ICC 1882 after digestion with *MseI* restriction enzyme. The PCR amplicons also correspond to the high and low RTR bulk along with another two parental lines (ICC 8261—high root trait ratio parent and ICC 283—low root trait ratio parent) of different mapping population.

CAPS/dCAPS markers represent cost‐effective assays to genotype SNPs in a segregating population. Therefore, to validate the identified five genic SNPs for 100SDW and six genic SNPs for RTR regions, a total of 11 CAPS/dCAPS primers were designed (Table S5). Of 11, eight primers amplified prominent fragments of expected size in two different parental combinations (ICC 4958 × ICC 1882; ICC 283 × ICC 8261). Of these eight primer pairs, six (three for each 100SDW and RTR trait) were found polymorphic using their respective restriction enzymes. Of six polymorphic markers, four Ca_04364_11311944 and Ca_04607_13822453 for 100SDW and Ca_04586_13666705 and Ca_04586_13666728 for RTR were validated in the high and low DNA pools. The fragment size of high DNA pools of 100SDW and RTR followed the similar pattern of the tolerant line ICC 4958 and the low DNA pool of 100SDW and RTR bulks followed the similar pattern of the sensitive line ICC 1882. The details on amplified fragment size and digested product size of each primer pairs are presented in Figures 3 and 4 and Table S6. Of 11 primer pairs tested, four CAPS markers follow the similar pattern in high trait parent and high bulk and similarly to low trait parent and low bulk. This ensures the utilization of these CAPS /dCAPS markers in marker‐assisted breeding programme.

### Confirmation of marker–trait associations

The genotyping data of four candidate CAPS/dCAPS markers (Ca_04364_11311944 and Ca_04607_13822453, Ca_04586_13666705 and Ca_04586_13666728) were used to develop genetic linkage map. As a result, a map length of 25.18 cM was obtained for linkage group 4. Single‐marker QTL analysis showed a high significance (*P* < 0.01%) for all four markers. The candidate markers for 100SDW, Ca_04364_11311944 and Ca_04607_13822453, explained high phenotypic variation of 28.61% with LOD score of 64.58 and 19.25% with LOD score of 45.61, respectively (Table 4). Similarly, the candidate markers for RTR, Ca_04586_13666728 and Ca_04586_13666705 explained a phenotypic variation of 23.29% with LOD score of 51.11 and 26.46% with LOD score of 56.33, respectively (Table 4).

**Table 4:** Single‐marker analysis for 100 seed weight (100SDW) and root trait ratio (RTR)

Marker	PVE	LOD	*P*‐value
100SDW			
Ca_04364_11311944	28.61	64.58	0^a^
Ca_04607_13822453	19.25	45.61	0^a^
RTR			
Ca_04493_13666728	23.29	51.11	0^a^
Ca_04586_13666705	26.46	56.33	0^a^

PVE, phenotypic variation explained.

a *P*‐value <0.0001.

## Discussion

Genetic mapping of QTLs for economically important traits is an important component of the development of new and elite lines in plant breeding programmes. Classic methods of QTL mapping involve genotyping of segregating mapping population with polymorphic markers identified between parents and use of genotyping data with phenotyping data sets for defining significant genomic regions for the target traits. However, identification of polymorphic markers between contrasting parents is time‐consuming and tedious task (Schneeberger and Weigel, 2011). For instance, during earlier studies, a total of 2717 (between ICC 4958 × ICC 1882) and 2410 (between ICC 283 × ICC 8261) markers including simple sequence repeats (SSRs), genic molecular markers (GMMs) and EST‐SSRs were screened on the parental lines of these two mapping populations. As a result, only 321 (ICC 4958 × ICC 1882, 11.81%) and 230 (ICC 283 × ICC 8261, 9.54%) markers were found to be polymorphic between the parental lines (Varshney *et al*., 2014a). The markers showed polymorphism between parents were comparatively very low in comparison with other crops, and the main reason behind this is due to the presence of limited genetic diversity in chickpea parental lines (Roorkiwal *et al*., 2014). Furthermore, reduction in genotyping cost with the advent of NGS technologies and availability of the chickpea genome ensures that the number of genomewide markers is not a limiting factor for trait mapping (Varshney *et al*., 2014b). Therefore, the recently proposed NGS‐based QTL‐seq approach, which is a cost‐effective and rapid method of trait mapping, was adopted in this study as it is a proven approach to identify the target genomic region in rice (Takagi *et al*., 2013). This approach avoids the tedious genotyping of a large population, and it has the capacity to identify shorter genomic regions associated with a trait of interest than classical QTL mapping approaches. QTL‐seq also has an advantage that the markers located within the identified candidate regions can be further used for fine mapping and cloning experiments. These markers can be converted into cost‐effective CAPS/KASPar markers for deployment in genomics‐assisted breeding programmes.

In this study, sequencing of both bulks (high RTR and low RTR bulk) identified a comparatively large number of SNPs (7318 total SNPs and 3792 homozygous SNPs), which were used for calculating genomewide SNP‐index. Analysis of SNPs located in this region revealed a candidate gene, *Ca_04586* coding for cytochrome P450 monooxygenase, an abscisic acid (ABA) 8′ hydrolase which involved in ABA catabolism (Krochko *et al*., 1998), reducing ABA during seed imbibition. Cytochrome P450 monooxygenase gene has been shown up‐regulated during drought stress in Arabidopsis and found to play an important role in the maintenance of ABA levels in plants (Kushiro *et al*., 2004). The possible role of *Ca_04586* in drought tolerance in chickpea needs to be investigated further.

Similarly for 100SDW, two genomic regions on CaLG01 (1.08 Mb) and CaLG04 (2.70 Mb) were identified. It is interesting to note that several studies reported the presence of QTL for 100SDW on CaLG04 in chickpea (Abbo *et al*., 2005; Cho *et al*., 2002; Cobos *et al*., 2007), showing the importance of this linkage group in yield trait improvement for chickpea. These results highlighted the significance of the QTL‐seq approach in identifying refined and reliable candidate regions for the trait of interest. Detailed analysis of SNP frequencies identified the gene *Ca_04607* on CaLG04, coding for a ‘transmembrane protein’. Role of transmembrane proteins in controlling grain weight, grain length, grain width and thickness has been earlier reported in rice (Fan *et al*., 2006; Shomura *et al*., 2008; Song *et al*., 2007). Based on these findings, it is possible to target *Ca_04607* for fine mapping and cloning of seed weight‐related genes in chickpea. However, it is important to note that this gene was not present in the prioritized list of genes in our recent study despite the mapping of 100SDW responsive QTLs in the same genomic region from 11.12 to 13.82 Mb on CcLG04 (Kale *et al*., 2015; 13.23–13.37 Mb for ‘*QTL‐hotspot‐a*’ and 13.39–13.54 for ‘*QTL‐hotspot‐b*’ on CaLG04). This might be due to low‐coverage sequencing of RIL population (average 0.72 X per RILs) or because of errors in genotyping of RILs or resequencing of parental genotypes.

Recently, Das *et al*. (2015) utilized QTL‐seq approach utilizing RILs population (ICC 7184 × ICC 15061) and reported major seed weight QTL on CaLG01 (CaqSW1.1; 0.83–0.87 Mb). This QTL region was further narrowed down using an integrated approach and reported CSN8 as a possible candidate gene for controlling seed weight in chickpea. Similarly, five robust QTLs on five different linkage groups (CaLG 1, 2, 5, 6 and 7) with PVE ranged from 10.07% to 22.31% using GBS approach in SBD377 × BGD112 mapping population (Verma *et al*., 2015). Thus, the genomic regions reported using similar approaches in earlier study are different from that we reported in this study, which are novel.

**Figure 5:**
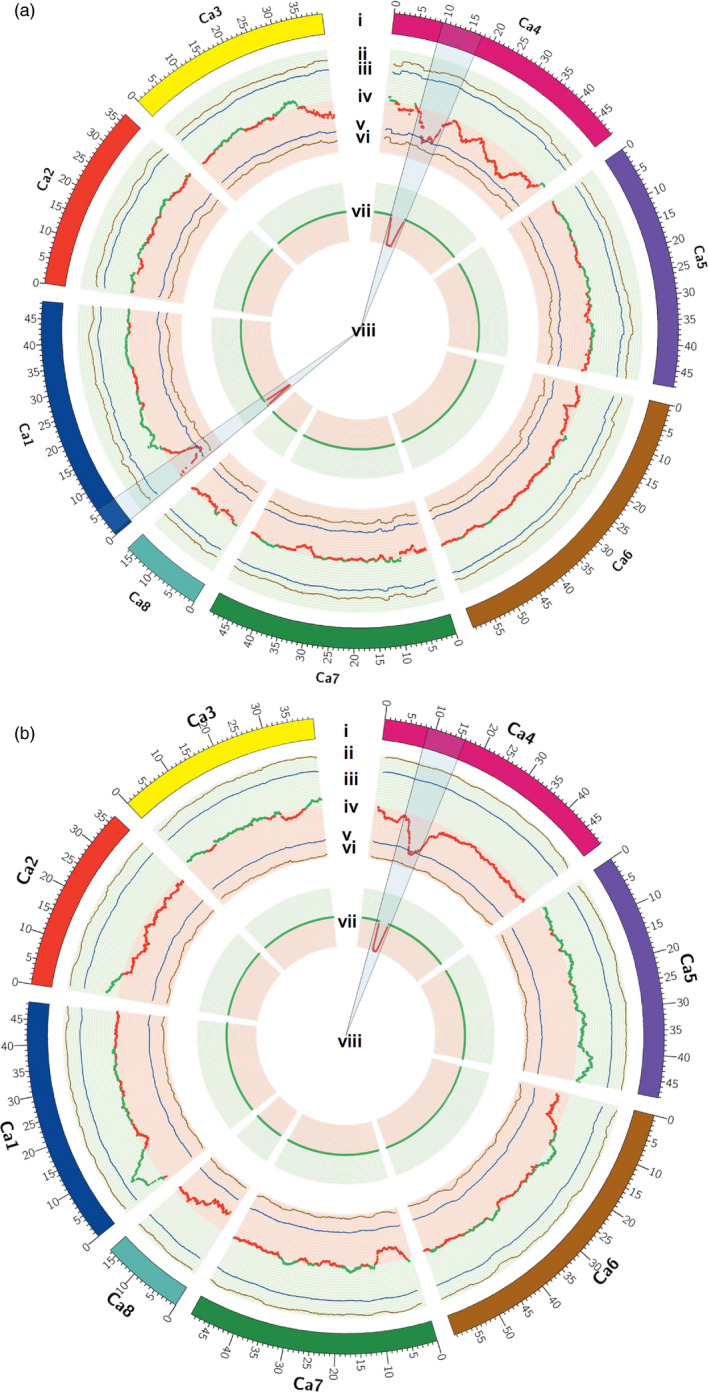
Co‐localization of QTLs from traditional and QTL‐seq approach for 100SDW and RTR. (a) Co‐localization of QTLs mapped for 100SDW through traditional and QTL‐seq method. (i) Psuedomolecules of reference genome CDC Frontier (Varshney *et al*., 2013), (ii) upper probability values at 99% confidence (*P* < 0.01), (iii) upper probability values at 95% confidence (*P* < 0.05), (iv) genomewide ΔSNP‐index [red dots denote ΔSNP‐index ranged from 0 to −1 and contributed by high trait parent (ICC 4958) and green dots denote ΔSNP‐index ranged from 0 to 1 and contributed by low trait parent (ICC 1882)], (v) lower probability values at 95% confidence (*P* < 0.05), (vi) lower probability values at 99% confidence (*P* < 0.01), (vii) physical position of earlier mapped QTL (Varshney *et al*., 2014a) for 100SDW through traditional mapping approach. The physical positions of QTL were estimated through blast of the flanking primers to the chickpea genome. (viii) Common genomic positions on linkage group 1 (CaLG01) and linkage group 4 (CaLG04) were observed through both the approaches. (b) Co‐localization of QTLs mapped for RTR through traditional and QTL‐seq method. (i) Psuedomolecules of reference genome CDC Frontier (Varshney *et al*., 2013), (ii) upper probability values at 99% confidence (*P* < 0.01) for declaring significant ΔSNP‐index, (iii) upper probability values at 95% confidence (*P* < 0.05) for declaring significant ΔSNP‐index, (iv) genomewide ΔSNP‐index [red dots denote ΔSNP‐index ranged from 0 to −1 and contributed by high trait parent (ICC 4958) and green dots denote ΔSNP‐index ranged from 0 to 1 and contributed by low trait parent (ICC 1882)], (v) lower probability values at 95% confidence (*P* < 0.05), (vi) lower probability values at 99% confidence (*P* < 0.01), (vii) physical position of earlier mapped QTL (Varshney *et al*., 2014a) for RTR through traditional mapping approach. The physical position of QTL was estimated through blast the flanking primers into the chickpea genome. (viii) Common genomic positions on CaLG04 were observed through both the approaches.

To understand the robustness and precision of identification of the genomic regions responsible for 100SDW and RTR over the classical QTL mapping studies, we compared the results of this work with our earlier studies (Jaganathan *et al*., 2015 and Varshney *et al*., 2014a). In the case of 100SDW, Varshney *et al*. (2014a) reported two major QTLs one each on CaLG01 and CaLG04 that explained 10.31% and 58.20% of phenotypic variation, respectively. Based on the physical position of flanking markers, the QTLs for 100SDW spanned 6.57 Mb (2.93–9.51 Mb) on CaLG01 and 6.75 Mb (10.07–16.83 Mb) on CaLG04, while Jaganathan *et al*. (2015) reported two major QTLs one each on CaLG01 and CaLG04 that explained 16.23% and 60.41% of phenotypic variation, respectively. Based on the physical position of flanking markers, the QTLs for 100SDW spanned 8.43 Mb (1.08–9.51 Mb) on CaLG01 and 0.45 Mb (13.68–14.14 Mb) on CaLG04. While comparing this study with previous studies, the genomic region identified on CaLG01 was narrowed down from 6.57 Mb (Varshney *et al*., 2014a) and 8.43 Mb (Jaganathan *et al*., 2015) to 1.08 Mb (3.07 to 4.15 Mb) in the current study (Table S7 and Figure 5a), whereas genomic region identified on CaLG04 in the present study was 2.70 Mb in comparison with 6.75 Mb (Varshney *et al*., 2014a) and 0.45 Mb (Jaganathan *et al*., 2015) identified earlier.

Similarly, in the case of RTR, a major QTL (*QR3rtr01*) explaining 16.67% of phenotypic variance was reported between TAA170 and NCPGR21 on CaLG04 (Varshney *et al*., 2014a). Based on the physical position of the flanking SSR markers, the identified QTL was found to be located at 10.07–14.14 Mb (4.06 Mb region) on the physical map. With a denser genetic map (1007 loci), the same genomic region was further delaminated to 3.76 Mb (10.07–13.84 Mb; Jaganathan *et al*., 2015). Nevertheless, the QTL‐seq approach deployed in the present study narrowed down the region further (1.1 Mb) and identified the genomic region responsible for RTR (Table S7 and Figure 5b). In this region, a promising candidate gene, cytochrome P450 monooxygenase was identified which is reported to be abscisic acid (ABA) 8′ hydrolase involved in ABA catabolism (Kushiro *et al*., 2004).

In addition to the identification of candidate genes located in genomic regions associated with 100SDW and RTR, SNP markers were developed that can be directly implemented in selection strategies in breeding programmes. Of 11 tested, four CAPS/dCAPS markers follow the similar pattern in the high trait parent and the high bulk and similarly to low trait parent and low bulk. Single‐marker analysis of these markers shows significant correlation with the traits measured, confirming their suitability for application in marker‐assisted selection. The current study suggests that WGRS‐based BSA mapping methods can be adopted for other traits of relevance in crop improvement, potentially avoiding cost and time constraints associated with traditional QTL mapping and providing a powerful means to rapidly refine genomic regions containing candidate genes.

## Experimental procedures

### Plant materials

Chickpea recombinant inbred line (RIL) population (ICCRIL03) comprising 262 lines developed from a drought tolerant genotype, ICC 4958 (with high 100SDW and high RTR) and a drought‐sensitive genotype, ICC 1882 (low 100SDW and low RTR) was used in the present study. Extensive phenotyping data under rainfed conditions for several drought component traits (20 traits) in 1–7 seasons and 1–5 locations along with marker data enabled by Varshney *et al*. (2014a) to provide greater insights into the drought tolerance and genomic regions responsible for the traits studied.

### Construction of pools

Extreme bulks were prepared for 100SDW and RTR traits based on precise phenotyping data obtained for 5 years (2005, 2006, 2007, 2008 and 2009) and 2 years (2005 and 2007), respectively, under rainfed conditions. For developing the extreme bulks for each trait, 15 RILs with high mean phenotypic values and 15 RILs with low mean phenotypic values were selected. The equimolar concentration of DNA from 15 RILs with high phenotypic values was pooled together as one bulk, and similarly DNA from low mean phenotypic values was pooled together as another bulk. Thus, four extreme bulks two each for both the traits were prepared for sequencing.

### Construction of libraries and Illumina sequencing

A total of five Illumina libraries (four from extreme bulks mentioned above and one from ICC 4958 the drought tolerant parent) were prepared using TruSeq DNA Sample Prep kit LT, (set A) FC‐121‐2001. Two microgram DNA from each sample was sheared using diagenode Bioruptor^®^ NGS (Diogenode, Liege, Belgium), end repaired and adapter ligated. Size selection of libraries was performed using 2% agarose gel to get a target insert size of 500–600 bp and purified for further analysis. Further, the libraries were enriched using adaptor compatible PCR primers. The size distribution of amplified DNA libraries was checked on an Agilent Technologies 2100 Bioanalyzer using a High Sensitivity chip. The DNA libraries were sequenced on Illumina MiSeq platform with MiSeq Reagent Kit v2 (500‐cycles) (Illumina Inc., San Diego, CA) to generate 250‐base pair‐end reads.

### Construction of reference‐based assembly

The statistics of generated sequencing reads was estimated using raspberry tool of NGS‐QCbox (Katta *et al*., 2015). Further, QTL‐seq pipeline (http://genome-e.ibrc.or.jp/home/bioinformatics-team/mutmap, developed by Iwate Biotechnology Research Center, Japan) was used for calculating SNP‐index. Briefly, the cleaned reads of donor parent (ICC 4958) were first aligned to the reference genome (CDC Frontier; Varshney *et al*., 2013) using inbuilt BWA aligner (Li and Durbin, 2009). Coval was used for postprocessing and filtering of the alignment files (Kosugi *et al*., 2013). The variants called were then used to develop reference‐based assembly of the donor parent (ICC 4958) by substituting the bases with confidence variants calls in the genome. The reads from high and low bulks for both the traits were then aligned, and variants were called for both the bulks against the developed assembly.

### Calculation of SNP‐index

SNP‐index for each SNP position was calculated for both the bulks as per Abe *et al*.(2012) using the formula:

SNP‐index (at a position) = count of alternate base/count of reads aligned.

The positions with read depth <7 in both the bulks and SNP‐index <0.3 in either of the bulks were filtered out for ∆SNP‐index calculation. ∆SNP‐index can be calculated by subtracting SNP‐index of low bulk from SNP‐index of high bulk. Only SNP positions with ∆SNP‐index = −1 (i.e. the allele called in high bulk was same as that of ICC 4958 while contrastingly different in low bulk) were considered as the causal SNPs responsible for the trait of interest. The possible effects of the identified SNPs were inferred using SnpEff v3.0 open source program (Cingolani *et al*., 2012).

### Validation of identified candidate SNPs

Five genic SNPs for 100SDW and six genic SNPs for RTR identified in the present study were validated on four genotypes (ICC 4958, ICC 1882, ICC 283 and ICC 8261) and four bulks (High and low bulks of RTR and 100SDW). For this purpose, the SNPs were converted into CAPS/dCAPS markers using dCAPS Finder 2.0 (Neff *et al*., 2002) for the development of cost‐effective gel‐based markers. Of 11 SNPs, seven SNPs were converted into CAPS and four SNPs were converted into dCAPS. The predicted CAPS and dCAPS candidates were amplified on parental genotypes (ICC 4958 and ICC 1882) and also amplified on the resistant and susceptible bulks of respective pools from 100SDW and RTR. PCR amplicons of each CAPS and dCAPS marker were subjected to digestion with their respective restriction enzymes. The restricted samples were checked on 2% agarose gel electrophoresis.

### Single‐marker analysis

To study the effect of identified candidate markers on the population, the RIL population (ICC 4958 × ICC 1882) was genotyped with all the four candidate CAPS/dCAPS markers. The genetic map was constructed using JoinMap V4.0 with the default parameters. Single‐marker analysis (SMA) was carried out using the map along with the phenotypic data by QTL Cartographer. The mean phenotyping data generated under rainfed conditions used for making the pool as described earlier for 100SDW and RTR were used for this analysis. Based on the p‐value, the marker was determined to control the trait of interest.

## Author's contributions

V.K.S., A.W.K. and D.J. performed most of the experiments; V.K. and A.C. generated sequence data; A.W.K., H.T. and R.T. performed QTL‐seq analysis; P.M.G. contributed genetic material; V.K.S., A.W.K., D.J., M.T., M.R., V.G., T.S., R.T. and R.K.V. analysed and interpreted the QTL‐seq data; D.J., V.K.S. and R.K.V performed validation of candidate SNPs; V.K.S., D.J., M.T., M.R., T.S., R.T. and R.K.V. wrote the manuscript; R.K.V. conceived, designed and supervised the study and finalized the manuscript. All authors read and approved the manuscript.

## Conflict of interest

The authors declare that they have no competing interests.

## Supporting information


**Figure S2** Frequency distribution of 100SDW of the 262 RILs derived from a cross between ICC 4958 and ICC 1882 for different years (2005, 2006, 2007, 2008, 2009 and average) in field conditions.
**Figure S3** Frequency distribution of RTR levels of the 262 RILs derived from a cross between ICC 4958 and ICC 1882 over two different years (2005 and 2007) with mean values in controlled conditions.
**Figure S4** (a) Based on the 2 year phenotyping of 262 RILs for RTR, a total of 15 RILs with high RTR and 15 with low RTR were used to develop high RTR and low RTR bulks, respectively. (b) Similarly, based on 5 years data of 100SDW a total of 15 RILs with high 100SDW and 15 with low 100SDW were used to develop high 100SDW and low 100SDW bulks.
**Figure S5** SNP‐index plots for eight chromosomes of high 100SDW bulked DNA.
**Figure S6** SNP‐index plots for eight chromosomes of low 100SDW bulked DNA.
**Figure S7** The Δ(SNP‐index) plot obtained by subtraction of high 100SDW SNP‐index from low 100SDW SNP‐index for RILs obtained from a cross between ICC 4958 and ICC 1882.
**Figure S8** SNP‐index plots for eight chromosomes of high RTR bulked DNA.
**Figure S9** SNP‐index plots for eight chromosomes of low RTR bulked DNA.
**Figure S10** The Δ(SNP‐index) plot obtained by subtraction of high RTR SNP‐index from low RTR SNP‐index for RILs obtained from a cross between ICC 4958 and ICC 1882.
**Table S1** Chromosome wise SNPs distribution between low and high 100SDW bulks.
**Table S2** Chromosome wise SNPs distribution between low and high RTR bulks.
**Table S3** List of putative SNPs identified for 100SDW based on SNP‐index values.
**Table S4** List of putative SNPs identified for RTR based on SNP‐index values.
**Table S5** List of primers used for validation of candidate genes for RTR and 100SDW.
**Table S6** List of primers successfully validated for 100SDW and RTR.
**Table S7** Comparison of the identified QTLs from QTL‐seq with earlier studies.

**Figure S1** QTL‐seq approach used in chickpea.
