# Supplementary material for: QTL‐seq for rapid identification of candidate genes for 100‐seed weight and root/total plant dry weight ratio under rainfed conditions in chickpea
Source: Plant Biotechnol J. 2016 May 26;14(11):2110–9. doi: 10.1111/pbi.12567 (PMC5095801; doi:10.1111/pbi.12567)
Supplement: Supplementary file 1 — Figure S2 Frequency distribution of 100SDW of the 262 RILs derived from a cross between ICC 4958 and ICC 1882 for different years (2005, 2006, 2007, 2008, 2009 and average) in field conditions. Figure S3 Frequency distribution of RTR levels of the 262 RILs derived from a cross between ICC 4958 and ICC 1882 over two different years (2005 and 2007) with mean values in controlled conditions. Figure S4 (a) Based on the 2 year phenotyping of 262 RILs for RTR, a total of 15 RILs with high RTR and 15 with low RTR were used to develop high RTR and low RTR bulks, respectively. (b) Similarly, based on 5 years data of 100SDW a total of 15 RILs with high 100SDW and 15 with low 100SDW were used to develop high 100SDW and low 100SDW bulks. Figure S5 SNP‐index plots for eight chromosomes of high 100SDW bulked DNA. Figure S6 SNP‐index plots for eight chromosomes of low 100SDW bulked DNA. Figure S7 The Δ(SNP‐index) plot obtained by subtraction of high 100SDW SNP‐index from low 100SDW SNP‐index for RILs obtained from a cross between ICC 4958 and ICC 1882. Figure S8 SNP‐index plots for eight chromosomes of high RTR bulked DNA. Figure S9 SNP‐index plots for eight chromosomes of low RTR bulked DNA. Figure S10 The Δ(SNP‐index) plot obtained by subtraction of high RTR SNP‐index from low RTR SNP‐index for RILs obtained from a cross between ICC 4958 and ICC 1882. Table S1 Chromosome wise SNPs distribution between low and high 100SDW bulks. Table S2 Chromosome wise SNPs distribution between low and high RTR bulks. Table S3 List of putative SNPs identified for 100SDW based on SNP‐index values. Table S4 List of putative SNPs identified for RTR based on SNP‐index values. Table S5 List of primers used for validation of candidate genes for RTR and 100SDW. Table S6 List of primers successfully validated for 100SDW and RTR. Table S7 Comparison of the identified QTLs from QTL‐seq with earlier studies. [file PBI-14-2110-s001.docx]

**Supplementary Information**

**QTL-seq for rapid identification of candidate genes underlying major QTLs for 100-seed weight and root / total plant dry weight ratio in chickpea**

Vikas K Singh^1^, Aamir W Khan^1^, Deepa Jaganathan^1,2^, Mahendar Thudi^1^, Manish Roorkiwal^1^, Hiroki Takagi^3^, Vanika Garg^1,2^, Vinay Kumar^1^, Anu Chitikineni^1^, Pooran M Gaur^1^, Ryohei Terauchi^3^, Tim Sutton^4,5^, Rajeev K Varshney^1,6,*^

^1^International Crop Research Institute for the Semi-Arid Tropics (ICRISAT), Hyderabad, 502 324, India

^2^Osmania University, Department of Genetics, Hyderabad, 500007, India

^3^Iwate Biotechnology Research Center, Kitakami, Iwate, 024-0003, Japan

^4^South Australian Research and Development Institute, Adelaide, 5001, Australia

^5^University of Adelaide, Australia and School of Agriculture, Adelaide, 5064, Australia

^6^School of Plant Biology and Institute of Agriculture, The University of Western Australia, Crawley, WA, Australia

***Corresponding author:** [r.k.varshney@cgiar.org](mailto:r.k.varshney@cgiar.org)

Tel: 0091 40 30713305; Fax: 0091 40 3071 3074/ 3075

**Figure S1.** **QTL-seq approach used in chickpea.** (a) Two contrasting parents namely ICC 4958 (donor parent) and ICC 1882 (recipient parent) were crossed to develop F_7_ RILs through single seed descent method. (b) Developed RILs were phenotyped for multi years for targeted traits (100SDW and RTR) (c) Two sets of RILs pools were selected for constructed of 100SDW (high SDW and low SDW) and RTR (high RTR and low RTR) pools. (d) Two sets of DNA bulks were constituted based on equimolar mixing of DNAs of individuals. (e) These two sets of DNA bulks along with the donor parent (ICC 4948) were applied to whole-genome re-sequencing for further identification of SNPs for calculation of SNP-index through QTL-seq pipeline. (f) The QTL-seq pipeline was used for identification of candidate genomic regions for both the traits. (g) Based on the Δ SNP-index values for individual bulks putatively linked candidate SNPs were identified (h) Based on the Δ SNP-index values SNPs in the identified candidate genes were selected and validated through development of CAPS/ dCAPS markers in parental lines and thereof bulks along with different set of parental lines.


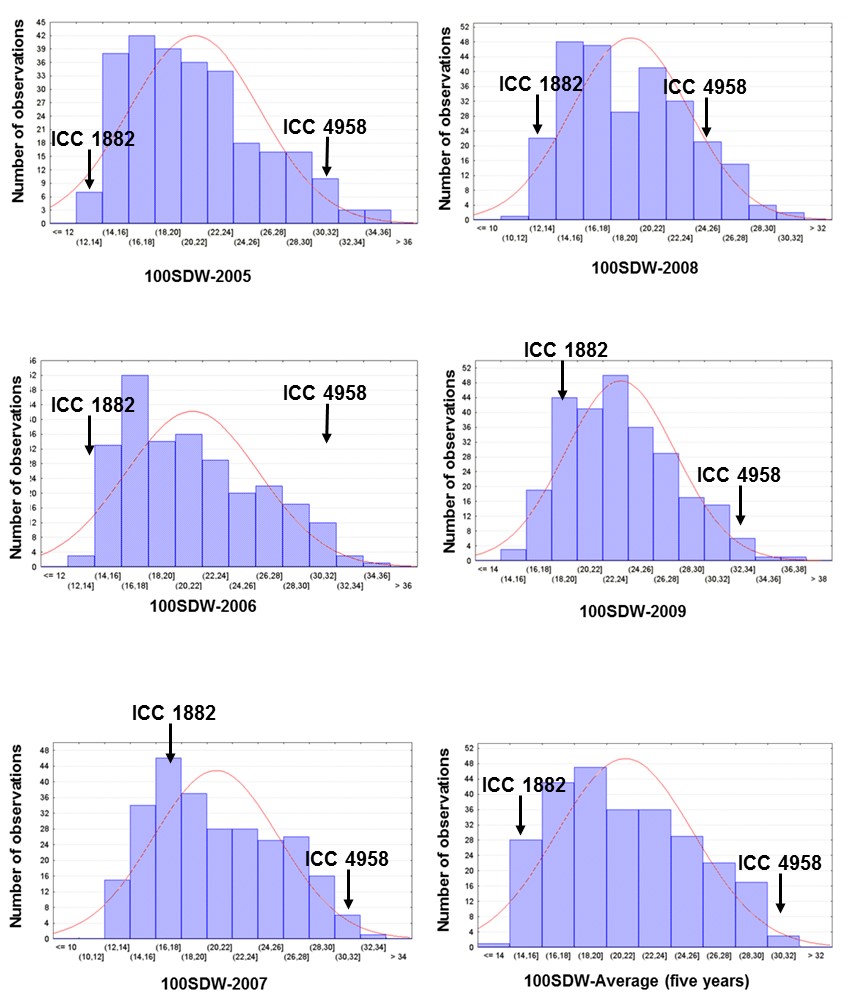


**Figure S2.** Frequency distribution of 100SDW of the 262 RILs derived from a cross between ICC 4958 and ICC 1882 for different years (2005, 2006, 2007, 2008, 2009 and average) in field conditions.


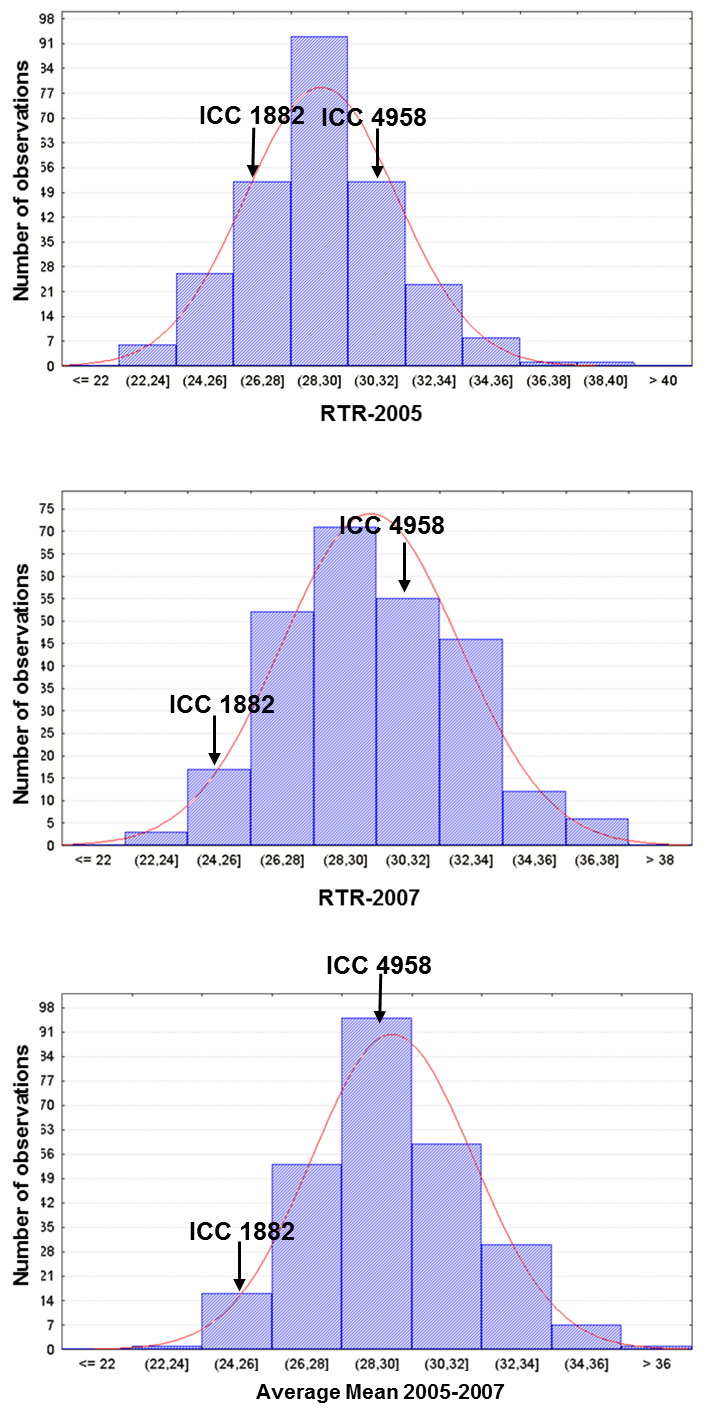


**Figure S3.** Frequency distribution of RTR levels of the 262 RILs derived from a cross between ICC 4958 and ICC 1882 over two different years (2005 and 2007) with mean values in controlled conditions.


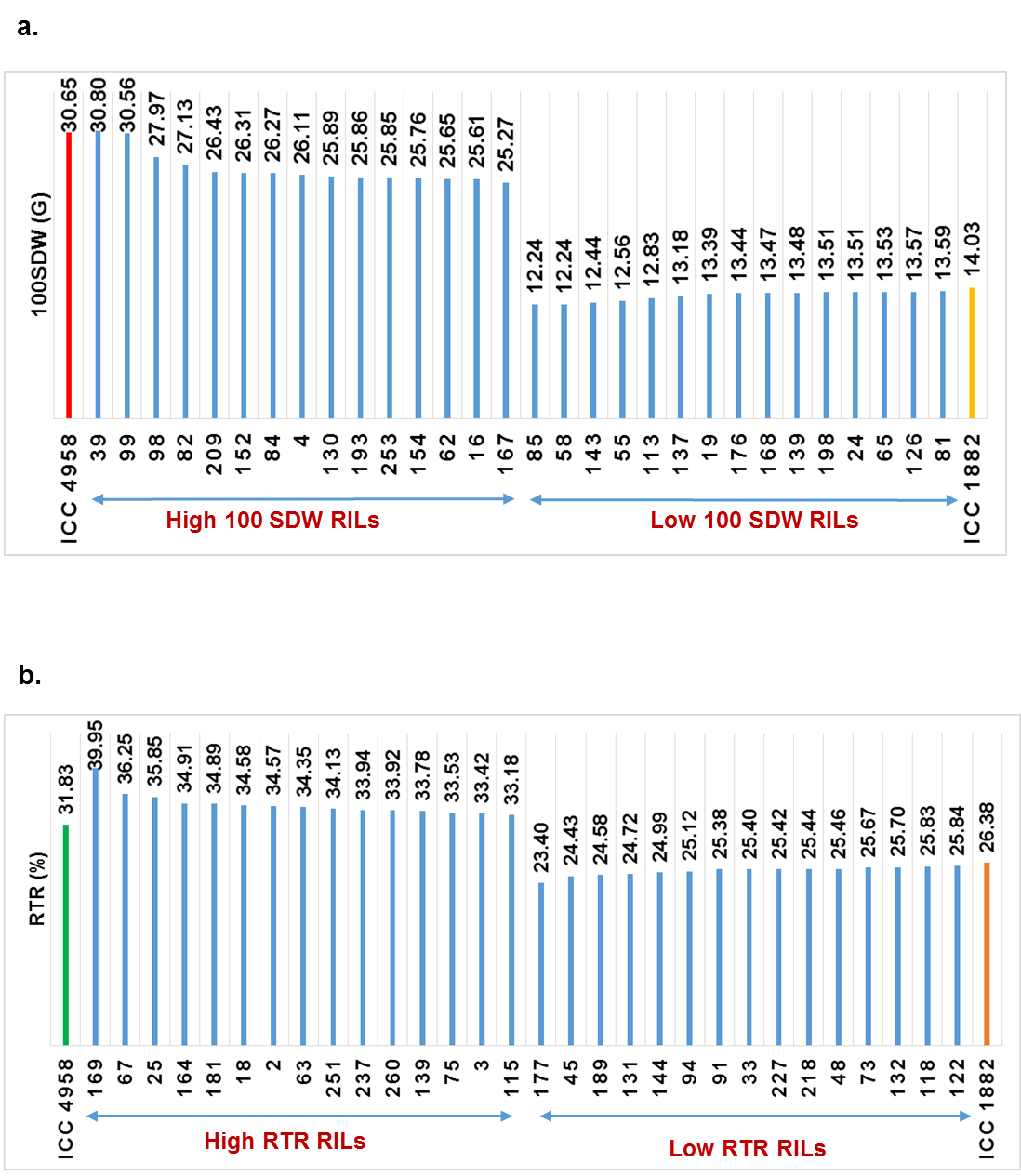


**Figure S4. (**a) Based on the five year phenotyping of 262 RILs for 100SDW, a total of 15 RILs with high 100SDW and 15 with low 100SDW were used to develop high 100SDW and low 100SDW bulks, respectively (b) Similarly, based on two years data of RTR a total of 15 RILs with high RTR and 15 with low RTR were used to develop high RTR and low RTR bulks.


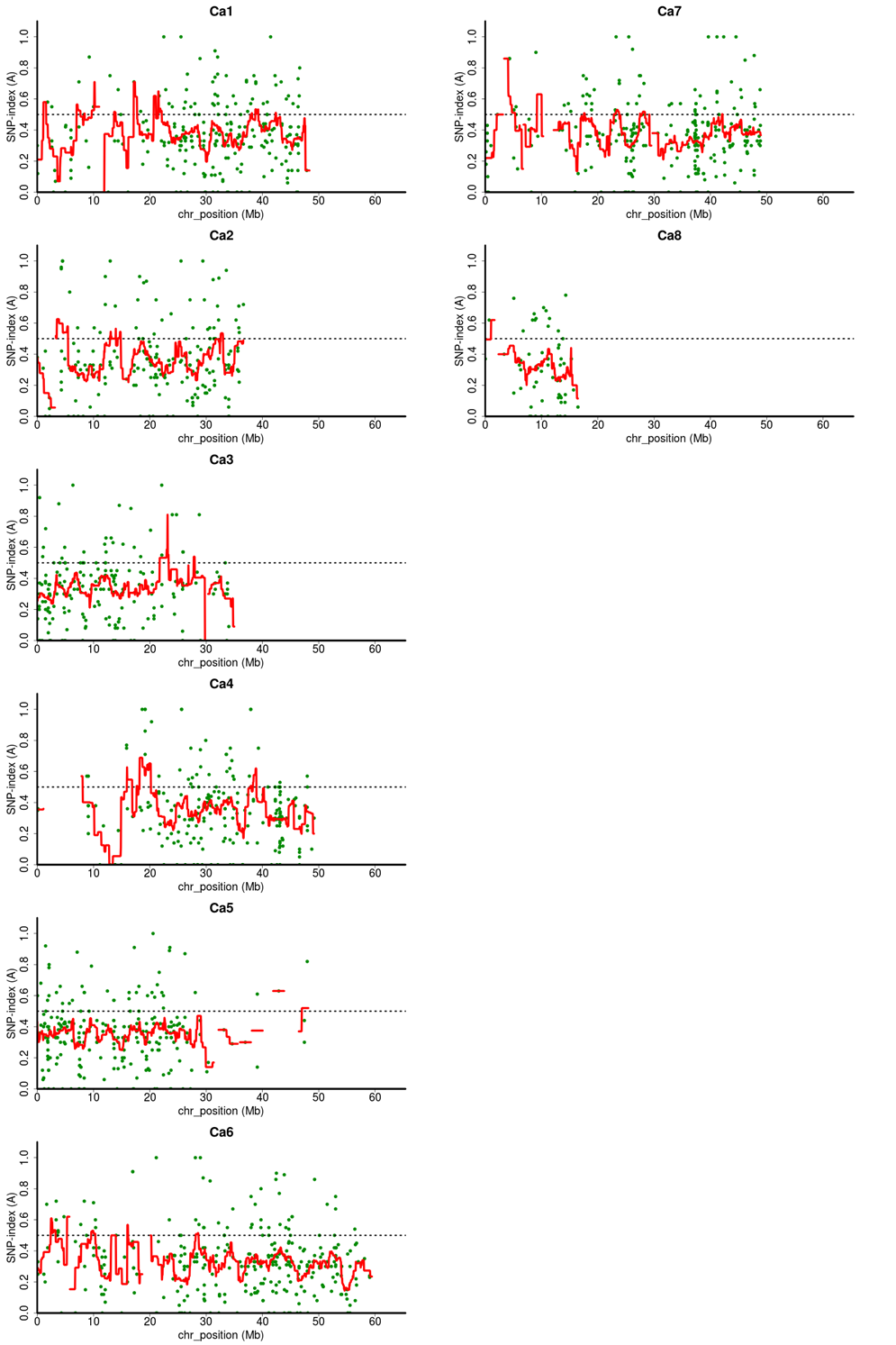


**Figure S5.** SNP-index plots for eight chromosomes of high 100SDW bulked DNA. Red lines indicate the sliding window average of 2 Mb interval with 10 kb increment for SNP-index.


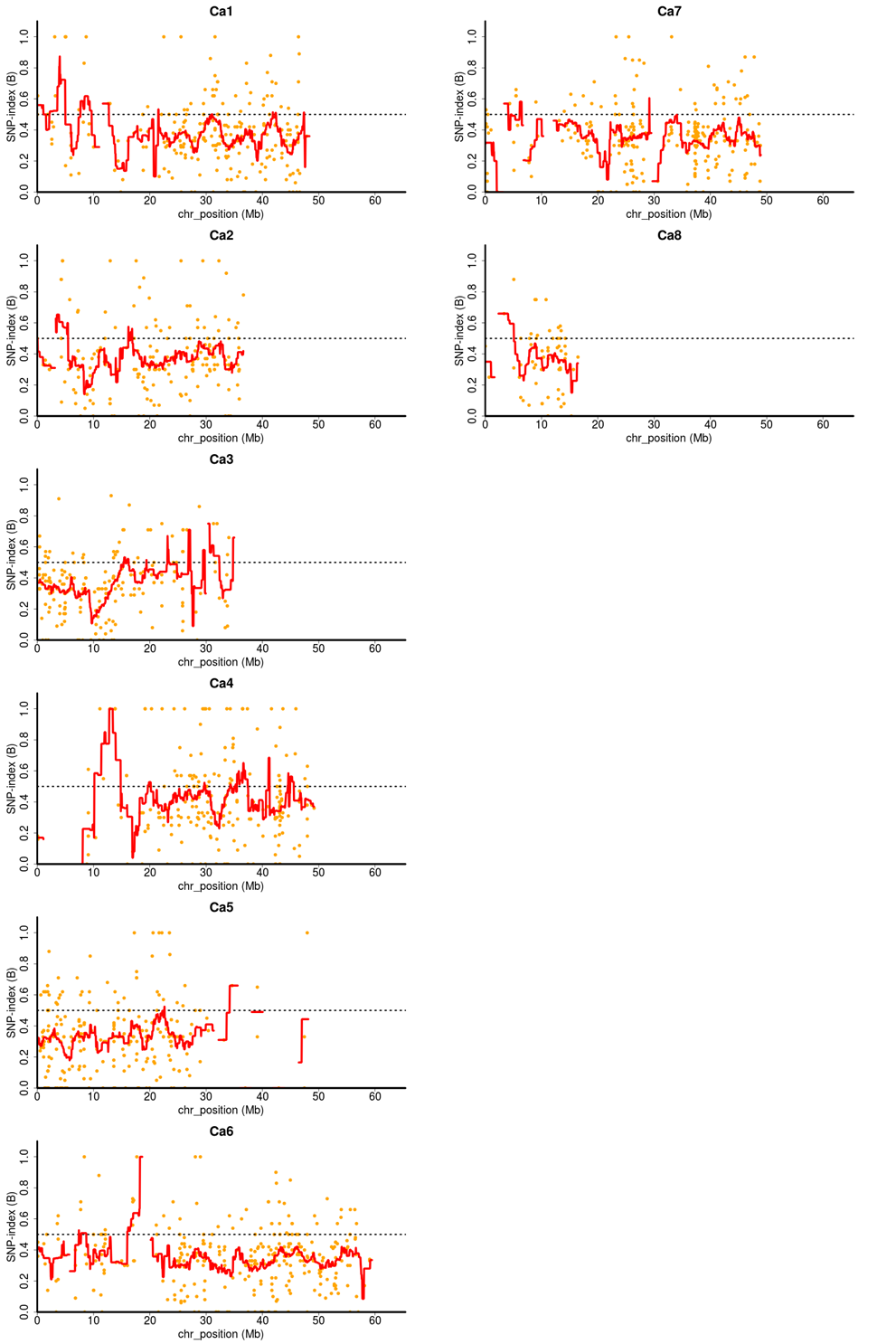


**Figure S6.** SNP-index plots for eight chromosomes of low 100SDW bulked DNA. Red lines indicate the sliding window average of 2 Mb interval with 10 kb increment for SNP-index.


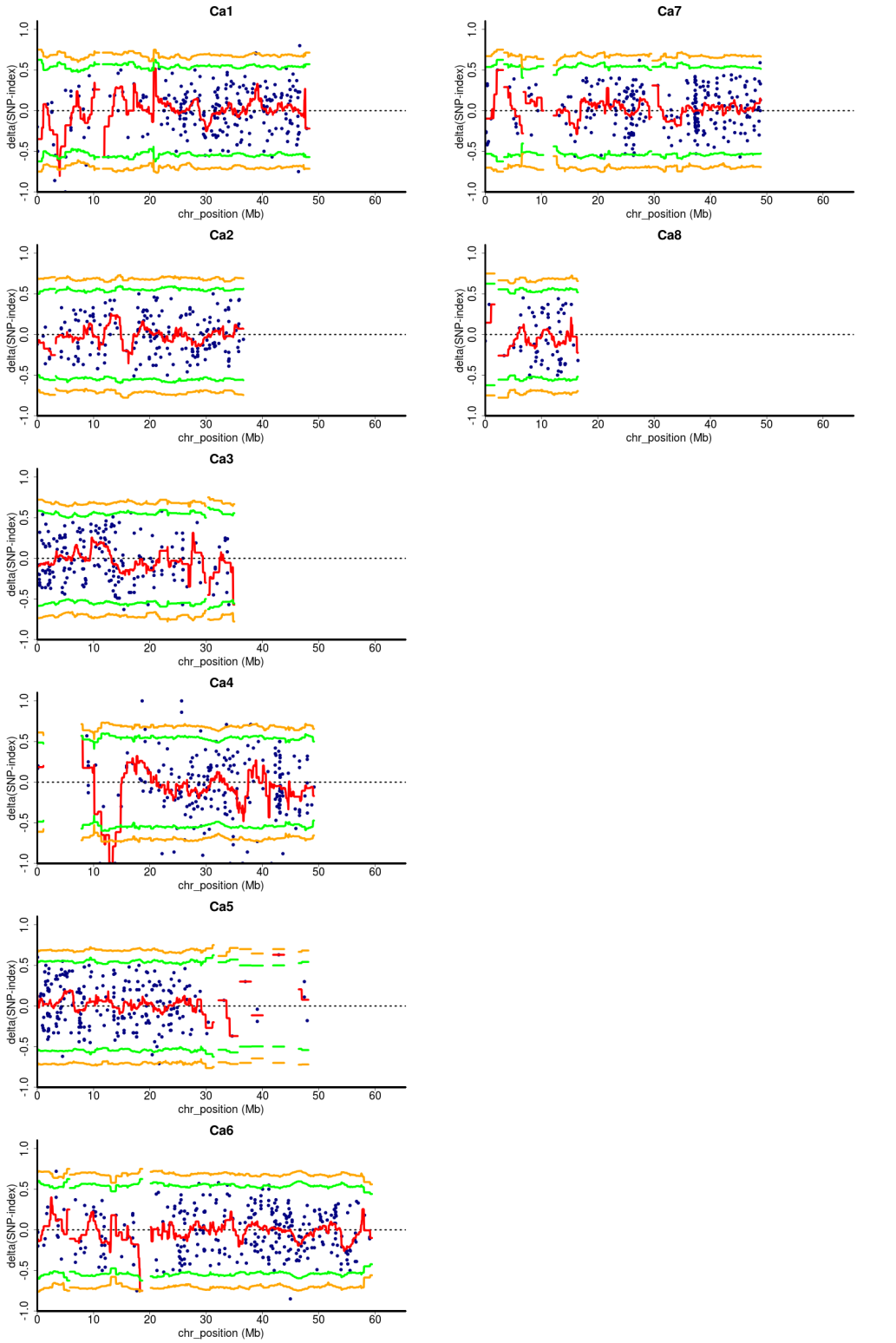


**Figure S7.** The Δ(SNP-index) plot obtained by subtraction of high 100SDW SNP-index from low 100SDW SNP-index for RILs obtained from a cross between ICC 4958 and ICC 1882. Statistical confidence intervals under the null hypothesis of no QTL are shown (green: P < 0.05; orange: P < 0.01).


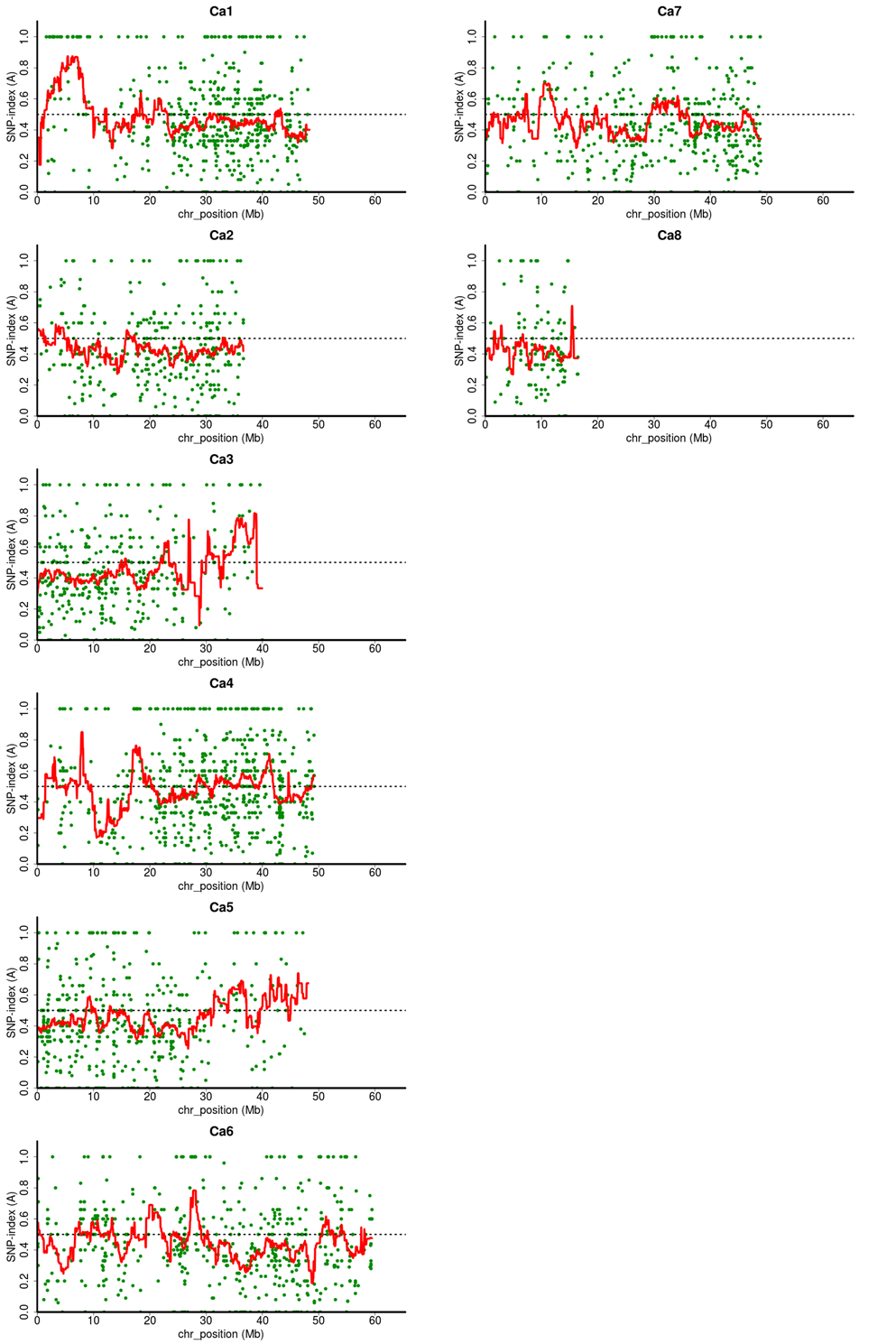


**Figure S8.** SNP-index plots for eight chromosomes of high RTR bulked DNA. Red lines indicate the sliding window average of the 2 Mb interval with 10 kb increment for SNP-index.


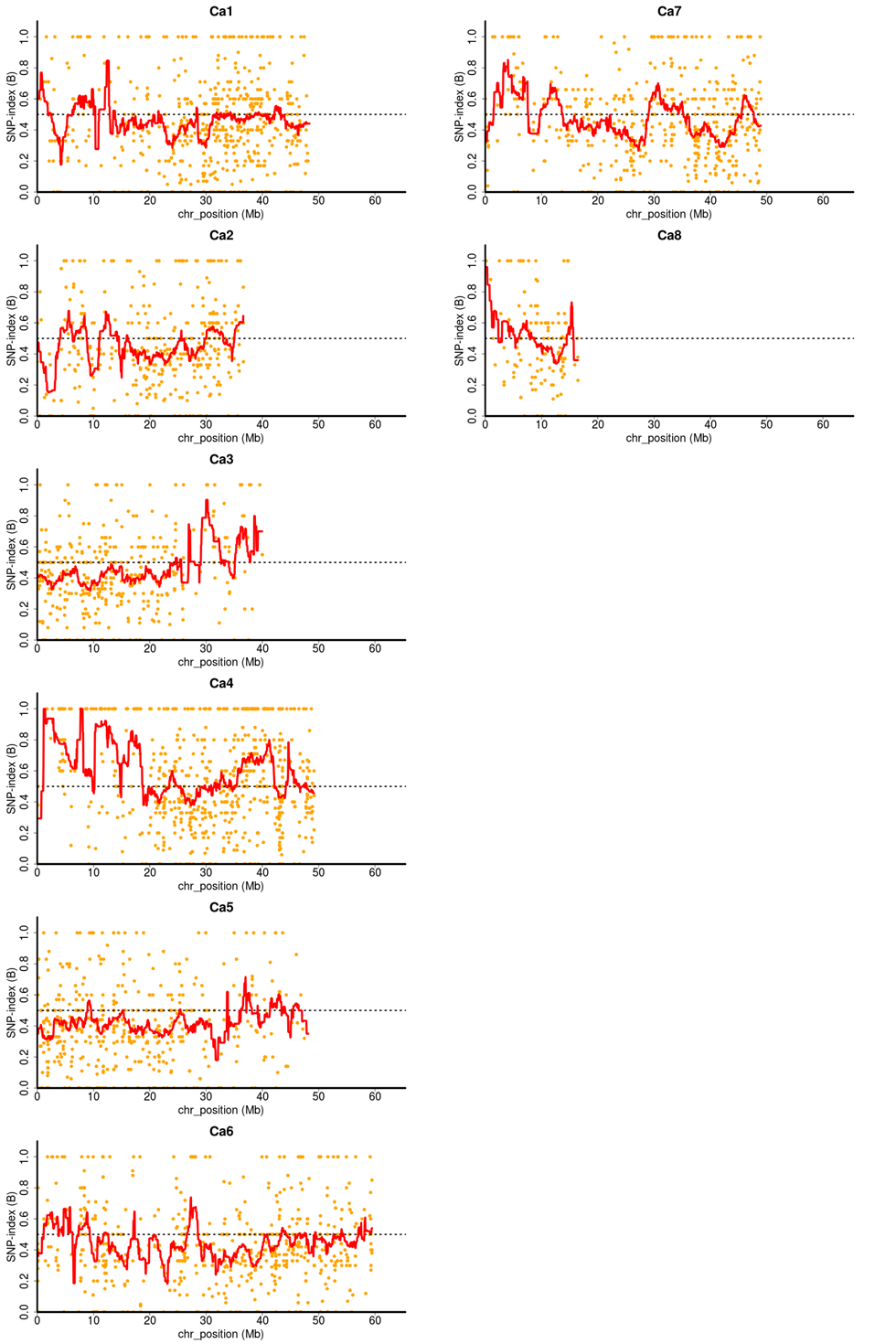


**Figure S9**. SNP-index plots for eight chromosomes of low RTR bulked DNA. Red lines indicate the sliding window average of 2 Mb interval with 10 kb increment for SNP-index.


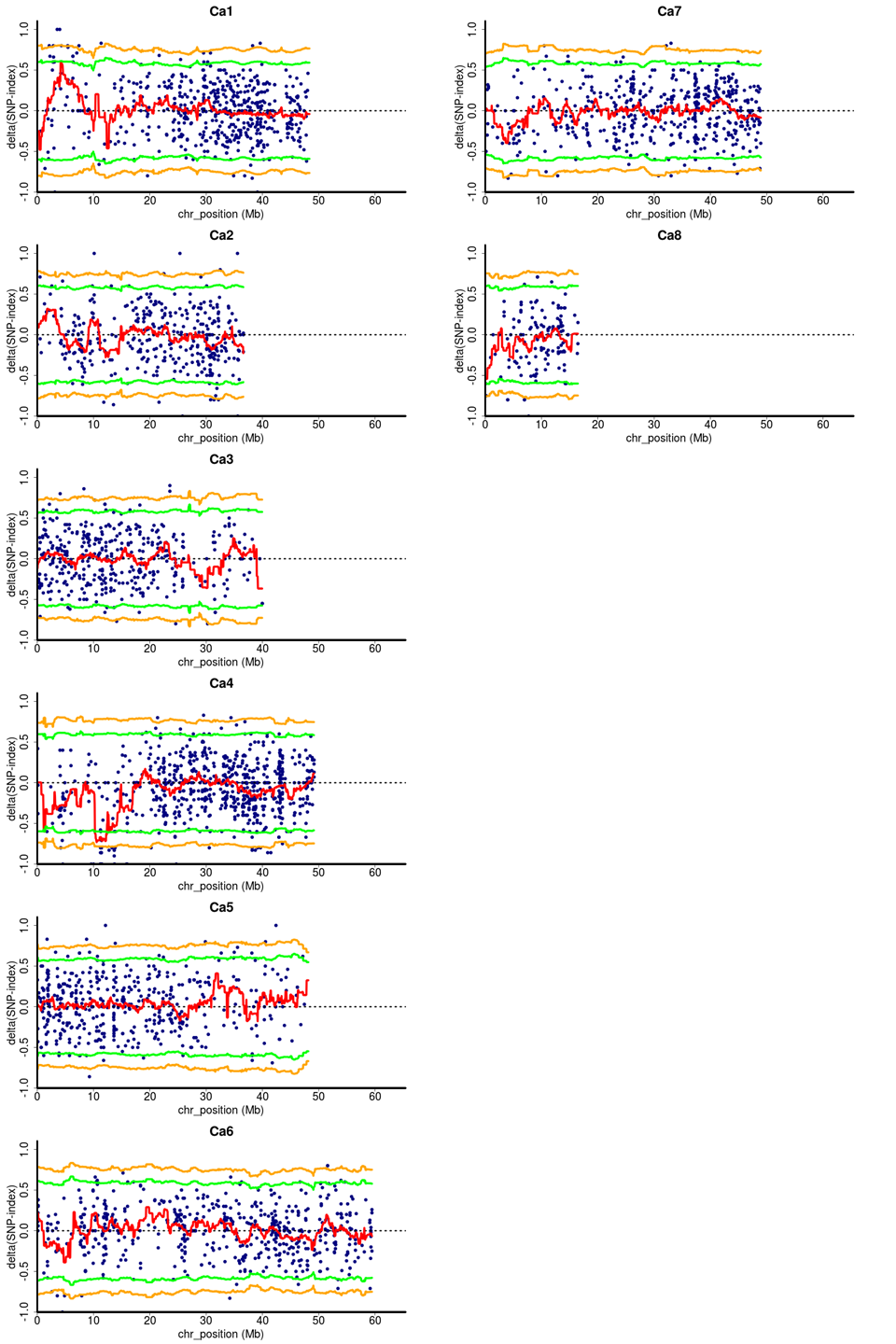


**Figure S10.** The Δ(SNP-index) plot obtained by subtraction of high RTR SNP-index from low RTR SNP-index for RILs obtained from a cross between ICC 4958 and ICC 1882. Statistical confidence intervals under the null hypothesis of no QTL are shown (green: *P* < 0.05; orange: *P* < 0.01).

**Table S1.** Chromosome wise SNPs distribution between low and high 100SDW bulks

| Linkage group | Total number of SNPs | Number of homozygous SNPs | Depth (X) range  in low 100SDW bulk | Depth range (X)  in high100SDW bulk |
| --- | --- | --- | --- | --- |
| CaLG01 | 964 | 238 | 5-70 | 5-74 |
| CaLG02 | 574 | 114 | 5-90 | 5-86 |
| CaLG03 | 593 | 125 | 5-72 | 5-64 |
| CaLG04 | 1024 | 438 | 5-71 | 5-70 |
| CaLG05 | 689 | 158 | 5-73 | 5-141 |
| CaLG06 | 1103 | 221 | 5-69 | 5-72 |
| CaLG07 | 881 | 189 | 5-74 | 5-93 |
| CaLG08 | 173 | 33 | 5-41 | 5-48 |
| Total | 6001 | 1516 |  |  |

**Table S2.** Chromosome wise SNPs distribution between low and high RTR bulks

| Linkage group | Total number of SNPs | Number of homozygous SNPs | Depth (X) range  in low RTR bulk | Depth range (X)  in high RTR bulk |
| --- | --- | --- | --- | --- |
| CaLG01 | 1282 | 608 | 3-58 | 3-52 |
| CaLG02 | 639 | 350 | 3-46 | 3-39 |
| CaLG03 | 704 | 360 | 3-52 | 3-48 |
| CaLG04 | 1848 | 1032 | 3-46 | 3-43 |
| CaLG05 | 666 | 365 | 3-41 | 3-33 |
| CaLG06 | 950 | 548 | 3-66 | 3-70 |
| CaLG07 | 1005 | 443 | 3-59 | 3-69 |
| CaLG08 | 224 | 86 | 3-38 | 3-30 |
| Total | 7318 | 3792 |  |  |

**Table S3.** List of putative SNPs identified for 100SDW based on SNP-index values

| Linkage group | Position (bp) | Reference allele  (ICA 4958) | Low 100SDW bulk allele | Read depth (X) | SNP-index (low bulk) |  | High 100SDWbulk allele | Read depth (X) | SNP-index (high bulk) |  | ΔSNP-index | Upper probability values at 99 % confidence (p<0.01) | Lower probability values at 99 % confidence (p<0.01) |
| --- | --- | --- | --- | --- | --- | --- | --- | --- | --- | --- | --- | --- | --- |
| CaLG01 | 3078766 | T | C | 5 | 1 |  | T | 6 | 0 |  | -1 | 0.80 | -0.80 |
| CaLG01 | 3104630 | G | T | 7 | 1 |  | G | 5 | 0 |  | -1 | 0.80 | -0.80 |
| CaLG01 | 3142432 | C | T | 5 | 1 |  | C | 8 | 0 |  | -1 | 0.80 | -0.80 |
| CaLG01 | 3727367 | A | G | 5 | 1 |  | A | 5 | 0 |  | -1 | 0.80 | -0.80 |
| CaLG01 | 4158149 | A | T | 10 | 1 |  | A | 6 | 0 |  | -1 | 0.83 | -0.83 |
| CaLG04 | 11128368 | T | G | 7 | 1 |  | T | 7 | 0 |  | -1 | 0.71 | -0.71 |
| CaLG04 | 11299562 | A | G | 5 | 1 |  | A | 6 | 0 |  | -1 | 0.80 | -0.80 |
| CaLG04 | 11299645 | T | C | 5 | 1 |  | T | 6 | 0 |  | -1 | 0.80 | -0.80 |
| CaLG04 | 11311944 | A | C | 5 | 1 |  | A | 6 | 0 |  | -1 | 0.80 | -0.80 |
| CaLG04 | 11353381 | C | T | 5 | 1 |  | C | 6 | 0 |  | -1 | 0.80 | -0.80 |
| CaLG04 | 11353412 | C | G | 6 | 1 |  | C | 5 | 0 |  | -1 | 0.80 | -0.80 |
| CaLG04 | 11449309 | C | T | 6 | 1 |  | C | 5 | 0 |  | -1 | 0.80 | -0.80 |
| CaLG04 | 12227513 | C | A | 5 | 1 |  | C | 5 | 0 |  | -1 | 0.80 | -0.80 |
| CaLG04 | 12299165 | G | A | 5 | 1 |  | G | 5 | 0 |  | -1 | 0.80 | -0.80 |
| CaLG04 | 12926479 | C | T | 11 | 1 |  | C | 5 | 0 |  | -1 | 0.80 | -0.80 |
| CaLG04 | 13095646 | G | A | 9 | 1 |  | G | 8 | 0 |  | -1 | 0.75 | -0.75 |
| CaLG04 | 13104366 | C | T | 5 | 1 |  | C | 5 | 0 |  | -1 | 0.80 | -0.80 |
| CaLG04 | 13665077 | A | C | 5 | 1 |  | A | 8 | 0 |  | -1 | 0.80 | -0.80 |
| CaLG04 | 13665097 | C | G | 5 | 1 |  | C | 8 | 0 |  | -1 | 0.80 | -0.80 |
| CaLG04 | 13694322 | G | A | 5 | 1 |  | G | 7 | 0 |  | -1 | 0.80 | -0.80 |
| CaLG04 | 13760326 | C | T | 5 | 1 |  | C | 5 | 0 |  | -1 | 0.80 | -0.80 |
| CaLG04 | 13780146 | C | G | 6 | 1 |  | C | 5 | 0 |  | -1 | 0.80 | -0.80 |
| CaLG04 | 13822383 | G | A | 7 | 1 |  | G | 7 | 0 |  | -1 | 0.71 | -0.71 |
| CaLG04 | 13822453 | A | G | 7 | 1 |  | A | 6 | 0 |  | -1 | 0.83 | -0.83 |
| CaLG04 | 13825787 | G | A | 7 | 1 |  | G | 5 | 0 |  | -1 | 0.80 | -0.80 |
| CaLG04 | 13825933 | T | G | 7 | 1 |  | T | 5 | 0 |  | -1 | 0.80 | -0.80 |

**Table S4.** List of putative SNPs identified for RTR based on SNP-index values

| Linkage group | Position (bp) | Reference allele  (ICC 4958) | Low RTRbulk allele | Read depth (X) | SNP-index (low bulk) |  | High RTRbulk allele | Read depth (X) | SNP-index (high bulk) |  | ΔSNP-index | Upper probability values at 99 % confidence (p<0.01) | Lower probability values at 99 % confidence (p<0.01) |
| --- | --- | --- | --- | --- | --- | --- | --- | --- | --- | --- | --- | --- | --- |
| CaLG04 | 12737206 | T | G | 3 | 1 |  | T | 3 | 0 |  | -1 | 1 | -1 |
| CaLG04 | 13053298 | T | A | 3 | 1 |  | T | 4 | 0 |  | -1 | 1 | -1 |
| CaLG04 | 13056600 | G | C | 4 | 1 |  | G | 4 | 0 |  | -1 | 1 | -1 |
| CaLG04 | 13158302 | G | A | 3 | 1 |  | G | 6 | 0 |  | -1 | 1 | -1 |
| CaLG04 | 13159332 | G | T | 8 | 1 |  | G | 4 | 0 |  | -1 | 1 | -1 |
| CaLG04 | 13183465 | T | C | 3 | 1 |  | T | 4 | 0 |  | -1 | 1 | -1 |
| CaLG04 | 13187696 | T | A | 3 | 1 |  | T | 3 | 0 |  | -1 | 1 | -1 |
| CaLG04 | 13361211 | T | G | 4 | 1 |  | T | 3 | 0 |  | -1 | 1 | -1 |
| CaLG04 | 13460421 | G | A | 3 | 1 |  | G | 4 | 0 |  | -1 | 1 | -1 |
| CaLG04 | 13481269 | T | A | 3 | 1 |  | T | 3 | 0 |  | -1 | 1 | -1 |
| CaLG04 | 13549638 | A | C | 3 | 1 |  | A | 3 | 0 |  | -1 | 1 | -1 |
| CaLG04 | 13593236 | G | T | 4 | 1 |  | G | 3 | 0 |  | -1 | 1 | -1 |
| CaLG04 | 13666705 | C | T | 3 | 1 |  | C | 3 | 0 |  | -1 | 1 | -1 |
| CaLG04 | 13666728 | C | T | 3 | 1 |  | C | 3 | 0 |  | -1 | 1 | -1 |
| CaLG04 | 13683889 | T | C | 3 | 1 |  | T | 5 | 0 |  | -1 | 1 | -1 |
| CaLG04 | 13684423 | A | G | 4 | 1 |  | A | 3 | 0 |  | -1 | 1 | -1 |
| CaLG04 | 13690423 | A | G | 5 | 1 |  | A | 8 | 0 |  | -1 | 0.8 | -0.8 |
| CaLG04 | 13695295 | C | G | 3 | 1 |  | C | 5 | 0 |  | -1 | 1 | -1 |
| CaLG04 | 13698500 | T | C | 4 | 1 |  | T | 5 | 0 |  | -1 | 1 | -1 |
| CaLG04 | 13708182 | T | C | 4 | 1 |  | T | 3 | 0 |  | -1 | 1 | -1 |
| CaLG04 | 13716902 | A | G | 5 | 1 |  | A | 7 | 0 |  | -1 | 0.8 | -0.8 |
| CaLG04 | 13743315 | T | A | 5 | 1 |  | T | 3 | 0 |  | -1 | 1 | -1 |
| CaLG04 | 13781245 | G | C | 3 | 1 |  | G | 4 | 0 |  | -1 | 1 | -1 |
| CaLG04 | 13796805 | G | A | 3 | 1 |  | G | 4 | 0 |  | -1 | 1 | -1 |
| CaLG04 | 13835549 | T | G | 3 | 1 |  | T | 5 | 0 |  | -1 | 1 | -1 |
| CaLG04 | 13835806 | C | A | 3 | 1 |  | C | 4 | 0 |  | -1 | 1 | -1 |

**Table S5**. List of primers used for validation of candidate genes for RTR and 100SDW

| Gene | SNP effect | SNP position | Marker name | Marker type | Enzyme^†^ | Primer sequence (5'- 3')^*^ | Product size | ICC 4958 × ICC 1882 | ICC 283 × ICC 8261 | High vs low bulk |
| --- | --- | --- | --- | --- | --- | --- | --- | --- | --- | --- |
| *100 SDW* | | | | | | | | | | |
| *Ca_04364* | Intron | 11311944 | Ca_04364_11311944 | CAPS | *AluI* | F-ATATGATGAGGGGTTGTGAA  R-TTTTGGTTTTCACTAAACAA | 493 | P | P | V |
| *Ca_04600* | Intron | 13760326 | Ca_04600_13760326 | CAPS | *MseI* | F- ATAGGCTGTAACGGCTAATG  R-GCATGACTAAACTCTTCTATGC | 506 | M | M | NT |
| *Ca_04602* | Intron | 13780146 | Ca_04602_13780146 | dCAPS | *BsrI* | F-CAGAGTTTCATTATTT**A**  R-CTGATCACTCAATCTTTTCTGT | 319 | NA | NA | NA |
| *Ca_04607* | Exon | 13822453 | Ca_04607_13822453 | dCAPS | *MseI* | F-GACTTATTTGATCTTATCTATT**A**  R-GGGTCTTGAACAATATATGAGC | 281 | P | P | V |
| *Ca_04607* | Intron | 13822383 | Ca_04607_13822383 | CAPS | *MaeII* | F-TTTTGCATAGAGTGATTGCT  R-TGTCATATGCTGTTTTGCAT | 205 | P | P | NV |
| *RTR* | | | | | | | | | | |
| *Ca_04493* | Intron | 12737206 | Ca_04493_12737206 | CAPS | *MseI* | F-CAAATGACAACAATTAGAAGAC  R-GTATTGCACGTTTTTCTTGC | 507 | NA | NA | NA |
| *Ca_04586* | Exon | 13666705 | Ca_04586_13666705 | dCAPS | *MseI* | F- GTTGAAGAGTCAAATATCT**T**  R- ACAAAATGGTACAAGCTCAA | 286 | P | P | V |
| *Ca_04586* | Exon | 13666728 | Ca_04586_13666728 | dCAPS | *MseI* | F- TCACATACTTAGAGGCA**T**  R- CTATTAGCAAATTGCAAACA | 302 | P | P | V |
| *Ca_04592* | Intron | 13708182 | Ca_04592_13708182 | CAPS | *NspI* | F- GACAAAAACTGGAGAAAAAC  R- TATCAGGACAAAAGGCAACT | 500 | P | P | NV |
| *Ca_04595* | Intron | 13716902 | Ca_04595_13716902 | CAPS | *ClaI* | F- AAAGTGACACTCATTGTGAGAC  R- ATGCTTCACTATAGCTTTCATC | 446 | M | M | NT |
| *Ca_04602* | Intron | 13781245 | Ca_04602_13781245 | CAPS | *TaqI* | F- CATCACTTTTTGCTTATTTG  R- GGGTACTTTTGTGCATGATT | 403 | NA | NA | NA |

^†^Enzymes used to digest the PCR amplified products

^*^Bold and underline fonts in primer sequences denotes altered bases for developing dCAPS markers

P: primer found polymorphism between ICC 4958 ×ICC1882 and ICC 283 × ICC 8261 during validation in 2% agarose gel

M: primer found monomorphic between ICC 4958 ×ICC 1882 and ICC 283 × ICC 8261 during validation in 2% agarose gel

NA: primer not amplified; V: primer found polymorphic (validated) between both the bulks in respect to their parents (RTR and 100 SDW); NV: primer not found polymorphic (not validated between both the bulks in respect to their parents (RTR and 100 SDW)

NT: primers not tested in bulks

**Table S6**. List of primers successfully validated for 100SDW and RTR

| Marker name | Trait | Marker type | Enzyme | ICC 4958  (High RTR and 100SDW parent) | | ICC 1882 (Low RTR and 100SDW parent) | | ICC 8261 (High RTR and 100SDW parent) | | ICC 283 (Low RTR and 100SDW parent) | | High- bulk^†^ | | Low- bulk^$^ | |
| --- | --- | --- | --- | --- | --- | --- | --- | --- | --- | --- | --- | --- | --- | --- | --- |
|  |  |  |  | Undigested (bp) | Digested (bp) | Undigested (bp) | Digested (bp) | Undigested (bp) | Digested (bp) | Undigested (bp) | Digested (bp) | Undigested (bp) | Digested (bp) | Undigested (bp) | Digested (bp) |
| Ca_04364_11311944 | 100SDW | CAPS | *AluI* | 500 | 500 | 500 | 240+  260 | 500 | 500 | 500 | 240+  260 | 500 | 500 | 500 | 240+  260 |
| Ca_04607_13822453 | 100SDW | dCAPS | *MseI* | 150 | 130+  20^*^ | 150 | 150 | 150 | 130+  20^*^ | 150 | 150 | 150 | 130+  20^*^ | 150 | 150 |
| Ca_04586_13666705 | RTR | dCAPS | *MseI* | 440 | 170+  150+  120 | 440 | 150+  120+  NV^*^ | 440 | 170+  150+  120 | 440 | 150+  120+  NV^*^ | 440 | 150+  120+  NV^*^ | 440 | 170+  150+  120 |
| Ca_04586_13666728 | RTR | dCAPS | *MseI* | 180 | 160+  20^*^ | 180 | 180 | 180 | 160+  20^*^ | 180 | 180 | 180 | 160+  20^*^ | 180 | 180 |

^*^NV: Not visible; PCR digested product with less than 50bp was not visible into the gels

^†^High- bulks represents High RTR bulk for RTR associated primers and High 100SDW bulk for 100SDW associated primers

^$^Low- bulks represents Low RTR bulk for RTR associated primers and Low 100SDW bulk for 100SDW associated primers

**Table S7.** Comparison of the identified QTLs from QTL-seq with earlier studies

| Linkage group | Trait | Varshney et al (2014) | | Jaganathan et al. (2015) | | QTL-seq (Current study) |
| --- | --- | --- | --- | --- | --- | --- |
|  |  | Physical position | PVE (%) | Physical position | PVE (%) | Physical position |
| CaLG01 | 100SDW | 2938353 - 9513885 (6.57 Mb) | 10.31 | 1083388 - 9513885 (8.43 Mb) | 16.23 | 3078766-4158149 (1.08 Mb) |
| CaLG04 | 100SDW | 10077153 - 16836157  (6.75 Mb) | 58.20 | 13687456 - 14146315 (0.45 Mb) | 60.41 | 11128368-13825933 (2.70 Mb) |
| CaLG04 | RTR | 10077153 - 14146315  (4.06 Mb) | 16.67 | 10077153 - 13840227 (3.76 Mb) | 13.56 | 12737206 -13835806 (1.10 Mb) |

Note: Values in parenthesis indicate the length of the genomic regions mapped during the study
