# Supplementary figures and images for: QTL‐seq for rapid identification of candidate genes for 100‐seed weight and root/total plant dry weight ratio under rainfed conditions in chickpea
Source: Plant Biotechnol J. 2016 May 26;14(11):2110–9. doi: 10.1111/pbi.12567 (PMC5095801; doi:10.1111/pbi.12567)

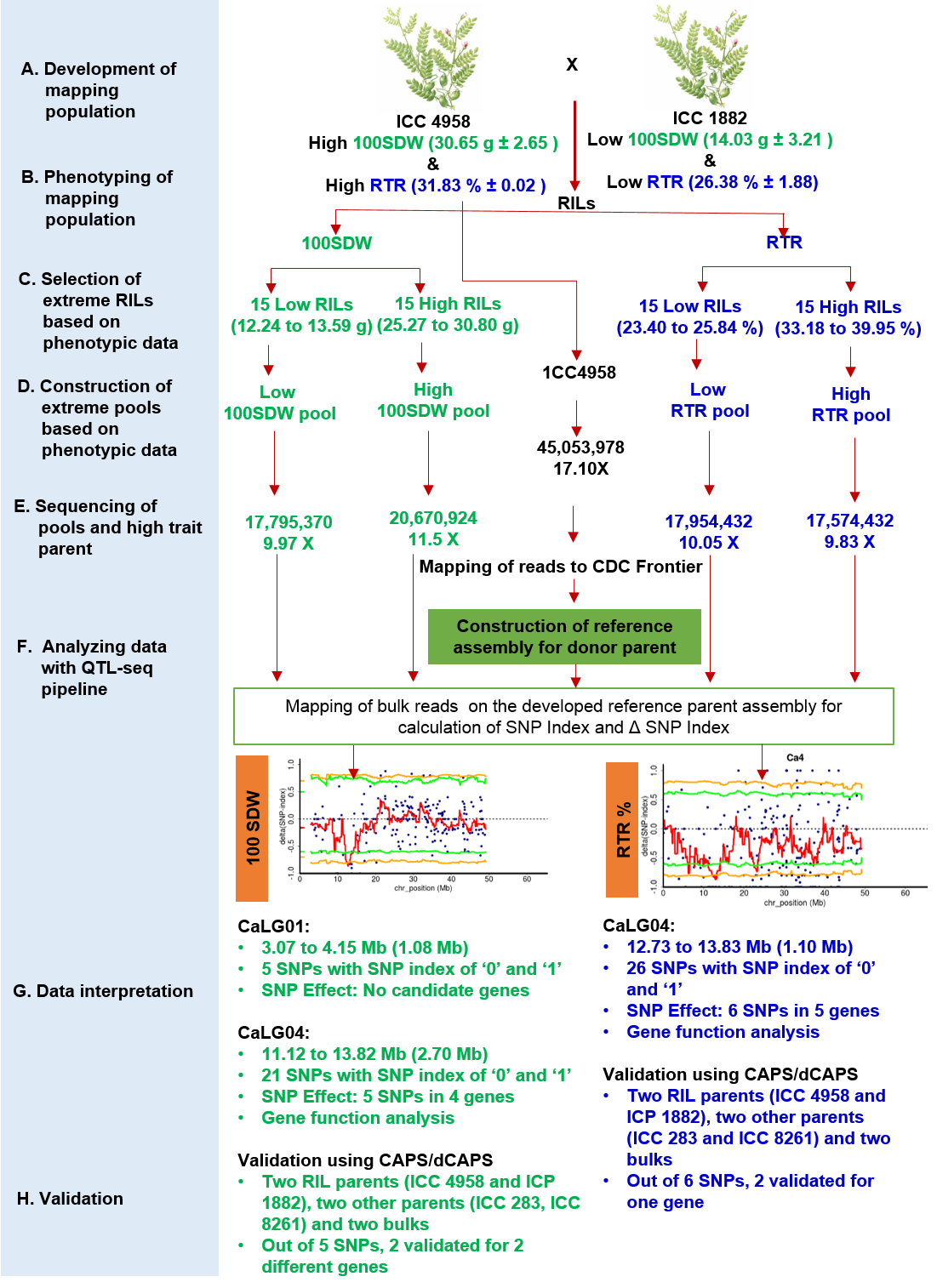

Supplement: Supplementary file 2 — Figure S1 QTL‐seq approach used in chickpea. [file PBI-14-2110-s002.tif]
